# UnifiedGreatMod: a new holistic modelling paradigm for studying biological systems on a complete and harmonious scale

**DOI:** 10.1093/bioinformatics/btaf103

**Published:** 2025-03-12

**Authors:** Riccardo Aucello, Simone Pernice, Dora Tortarolo, Raffaele A Calogero, Celia Herrera-Rincon, Giulia Ronchi, Stefano Geuna, Francesca Cordero, Pietro Lió, Marco Beccuti

**Affiliations:** Department of Computer Science, University of Turin, Via Pessinetto 12, Torino, 10149, Italy; Department of Computer Science, University of Turin, Via Pessinetto 12, Torino, 10149, Italy; Department of Computer Science, University of Turin, Via Pessinetto 12, Torino, 10149, Italy; Department of Molecular Biotechnology and Health Sciences, University of Torino, Via Nizza 52, Torino, 10126, Italy; Biomathematics Unit, Department of Biodiversity, Ecology and Evolution, Complutense University of Madrid, Madrid 28040, Spain; Department of Clinical and Biological Sciences, University of Torino, Regione Gonzole 10, Orbassano, 10143, Italy; Department of Clinical and Biological Sciences, University of Torino, Regione Gonzole 10, Orbassano, 10143, Italy; Department of Computer Science, University of Turin, Via Pessinetto 12, Torino, 10149, Italy; Department of Computer Science and Technology, University of Cambridge, Cambridge CB3 0FD, United Kingdom; Department of Computer Science, University of Turin, Via Pessinetto 12, Torino, 10149, Italy

## Abstract

**Motivation:**

Computational models are crucial for addressing critical questions about systems evolution and deciphering system connections. The pivotal feature of making this concept recognizable from the biological and clinical community is the possibility of quickly inspecting the whole system, bearing in mind the different granularity levels of its components. This holistic view of system behaviour expands the evolution study by identifying the heterogeneous behaviours applicable, e.g. to the cancer evolution study.

**Results:**

To address this aspect, we propose a new modelling paradigm, UnifiedGreatMod, which allows modellers to integrate fine-grained and coarse-grained biological information into a unique model. It enables functional studies by combining the analysis of the system’s multi-level stable states with its fluctuating conditions. This approach helps to investigate the functional relationships and dependencies among biological entities. This is achieved, thanks to the hybridization of two analysis approaches that capture a system’s different granularity levels. The proposed paradigm was then implemented into the open-source, general modelling framework GreatMod, in which a graphical meta-formalism is exploited to simplify the model creation phase and R languages to define user-defined analysis workflows. The proposal’s effectiveness was demonstrated by mechanistically simulating the metabolic output of *Escherichia coli* under environmental nutrient perturbations and integrating a gene expression dataset. Additionally, the UnifiedGreatMod was used to examine the responses of luminal epithelial cells to *Clostridium difficile* infection.

**Availability and implementation:**

GreatMod https://qbioturin.github.io/epimod/, epimod_FBAfunctions https://github.com/qBioTurin/epimod_FBAfunctions, first case study *E. coli*  https://github.com/qBioTurin/Ec_coli_modelling, second case study *C. difficile*  https://github.com/qBioTurin/EpiCell_CDifficile.

## Graphical abstract

**Graphical Abstract:**
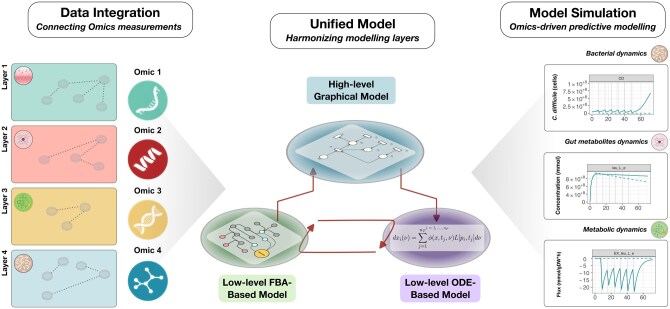

## 1 Introduction

Traditionally, biological problems have been approached using a reductionist perspective by thoroughly dissecting biological phenomena into individual components. However, with the advent of deep sequencing technologies, there has been an unprecedented opportunity to comprehensively characterize biological components simultaneously and gain a global sense of the systemic interactions that occur within a cell (Feist *et al.* 2009).

In computational biology, the pursuit of understanding complex biological systems has led to the development of diverse modelling approaches, each offering unique rationales and challenges. Constraint- and mechanism-based modelling have emerged as powerful tools for interpreting biological phenomena. Specifically, constrain-based approaches, such as Flux Balance Analysis (FBA) (Machado and Herrgård 2014, Opdam *et al.* 2017), can be employed to reconstruct genome-scale metabolic networks incorporating curated and systematized information about the known small metabolites and metabolic reactions of a cell type based on transcriptomics, proteomics, or metabolomics data (Bordbar *et al.* 2014). Furthermore, many studies have been trying to predict fluxes via transcriptomics and FBA or other constraint-based modelling techniques; recent approaches, such as Damiani *et al.* (2019), Di Filippo *et al.* (2021), and Galuzzi *et al.* (2022), map from expression profile to constraints for metabolic fluxes adjusting fluxes based on gene expression levels.

Among the available tools for FBA, the COBRA Toolbox is integral to the constraint-based modelling community, which focuses on open-source genome-scale metabolic models (GEMs). Noteworthy projects for high-throughput GEM generation include Path2Models (Büchel *et al.* 2013), AGORA (Magnúsdóttir *et al.* 2017), CarveMe (Machado *et al.* 2018), and BiGG Models (Norsigian *et al.* 2020). Moreover, human and microbial models and maps are available at the Virtual Metabolic Human website (Noronha *et al.* 2019), which collects human and gut metabolism data and links this information with nutrition.

These modelling approaches are efficient for the analysis of large-scale metabolic networks, but need detailed descriptions of molecular mechanisms, such as quantitative information, on molecular abundances and reaction kinetics. In contrast, fine-grained biological information is often modelled using systems of ordinary differential equations (ODEs), namely, mechanism-based approaches, to reproduce the dynamics of the system (Kendall *et al.* 1999). Although these approaches have the potential to reproduce reality more closely by investigating a biological phenomenon at a greater depth, they need help with scalability and parameterization issues, as they require a considerable amount of preliminary quantitative information, which limits their applicability to larger systems.

Recognizing the complementary nature of these approaches, there is growing interest in developing computational models that bridge the gap between constraint-based and mechanistic modelling paradigms.

An attempt to expand the possibilities of FBA is Dynamic Flux Balance Analysis (DFBA) proposed in Maranas and Zomorrodi (2016), Mahadevan *et al.* (2002), and Yang *et al.* (2019). DFBA represents a compromise between fully dynamic models—which cannot be simulated on a large scale—and steady state models—in which metabolite fluxes within a biological system remain constant over time and do not involve reaction kinetics (Palsson 2011, Maranas and Zomorrodi 2016). Its novelty lies in its ability to model metabolism under dynamic conditions, combining extracellular dynamics with intracellular steady states.

However, while DFBA allows investigating genome-scale networks under transient conditions, it cannot implement mechanistic knowledge of non-metabolic processes.

Recently, a collective effort has been to hybridize mechanism-based and constraint-based approaches. The goal is to tackle their limitations and merge their capabilities to decrease costs, improve efficiency, and perform more descriptive phenotype predictions.

A first insight was provided in Pernice *et al.* (2020a), where the authors proposed some initial indications on how this hybridization can be carried out through a high-level graphical formalism. In particular, the paper clearly shows how FBA can be exploited as a global source and sink of the system for specific sets of metabolites. Moreover, it discusses the possibility of using FBA in the network sub-models where there are missing kinetics and/or sloppy parameters and to close the original system.

Another notable example was the recent proof of concept of a hybrid ODE- and constraint-based model proposed in Ben Guebila and Thiele (2021), where an ODE-based glucose–insulin model was combined with a whole-body organ-resolved reconstruction of human metabolism to investigate Type 1 diabetes mellitus. However, the computational methodology and associated framework used in such work are peculiar to the example proposed.

Thus, to the best of our knowledge, a well-formalized methodology that is sufficiently general is still missing. To address such a gap in this work, we advance the hybridization of these two approaches by first formally defining the coupling between ODEs and FBA using a graphical meta-formalism and second by implementing this new modelling paradigm, namely ‘UnifiedGreatMod’, into the well-known open-source and general modelling framework *GreatMod* (Castagno *et al.* 2020) with the aim of providing a general-purpose, scalable, reproducible, and easy-to-use modelling tool, allowing researchers to study biological systems on a complete and harmonious scale.

Finally, it is important to emphasize that our framework is designed to work synergistically with the COBRA Toolbox, enabling users to seamlessly switch between or simultaneously utilize mechanistic-based and constraint-based models as needed. This interoperability ensures that users can effectively integrate existing COBRA models and tools with the models defined in ‘UnifiedGreatMod’, creating a cohesive and powerful modelling environment.

## 2 Methods

The flow of the paradigm involves the integration of heterogeneous data, considering different biological domains of a cell (such as genomics, transcriptomics, and metabolics data) or as distinct entities interacting with each other, including microbes interacting with host cells, facilitating their interchange within a holistic view of biological systems.

The harmonization, a central aspect of our paradigm, facilitates a dialogue among biological data and merges the computational aspects of the paradigm. Specifically, how the combination of mechanism and constraint-based models simulates the entire system to yield temporal evolution of key biological entities. This simulation enables the derivation of new insights into the systems under study.

**Figure 1.**
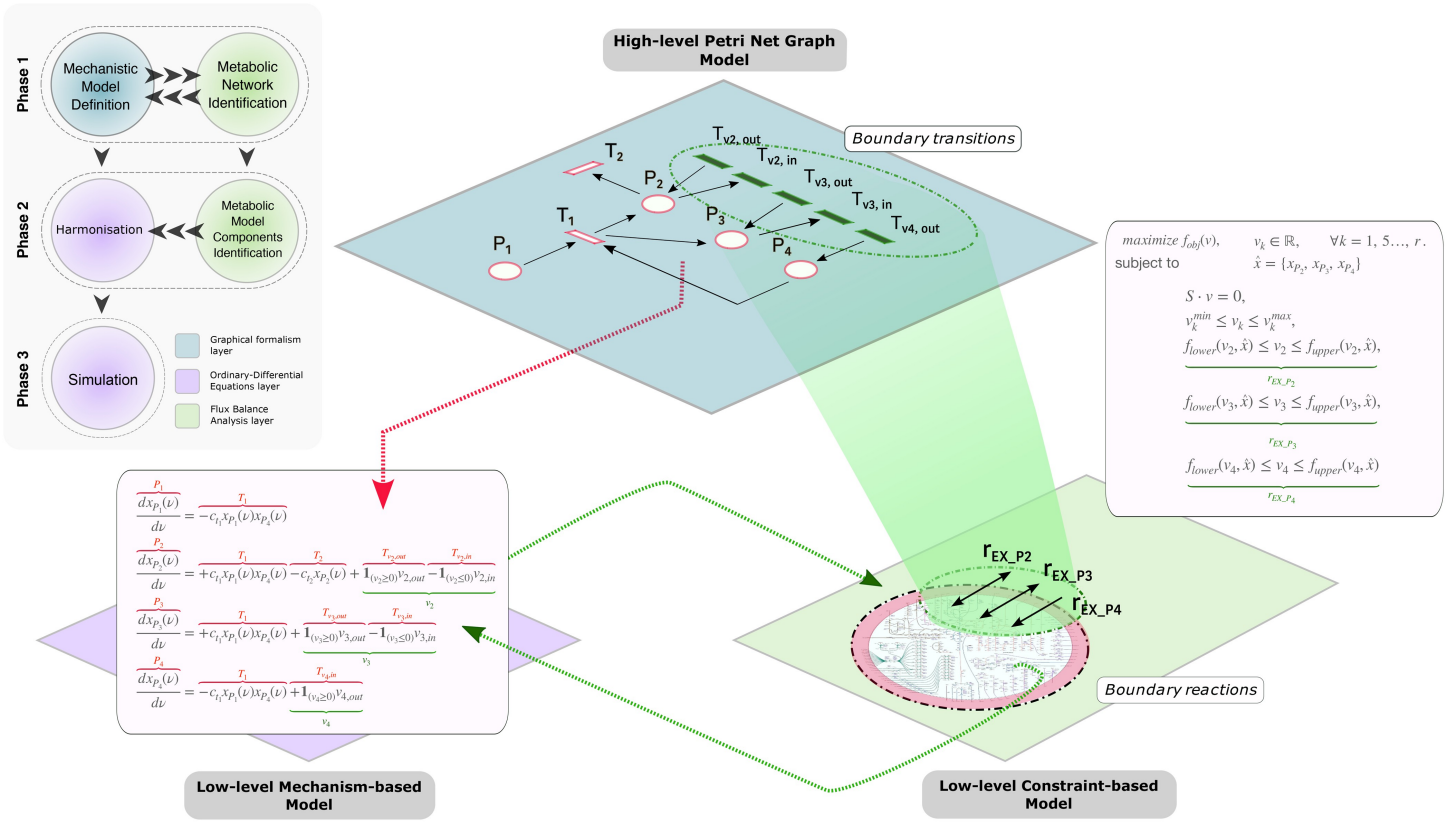
A sketch of the proposed modelling paradigm based on the graphical Petri Net formalism (i.e. ESPN) for stating the harmonization between the mechanistic-based and the constrained-based models. The red colour is associated with the components of the ODEs derived from the ESPN, while the green one with the fluxes obtained from the FBA.

‘UnifiedGreatMod’ aims to harmonize two different solution techniques: ODEs and FBA. This is achieved through a graphical formalism, which allows the modeller to state (i) the dynamic model represented by an ODEs system, (ii) the metabolic model studied by the FBA, and (iii) the coupling between the dynamic and metabolic models (see Fig. 1).

### 2.1 Overview of the coupling definition

The innovative contribution of ‘UnifiedGreatMod’ regards the harmonization between the dynamic and metabolic models. Intuitively, the key to this harmonization is the identification of ‘metabolites’ and ‘reactions’ to be coordinated, assuming (i) the existence of a set of metabolites shared between the two models, representing the resources that might be produced or consumed by both models, and (ii) the existence of boundary reactions that allow the sharing of the respective metabolite between the models. The following describes the idea behind the harmonization step, while all the theoretical details are provided in the Supplementary Material S1.1.

As shown in the left-upper corner of Fig. 1, the ‘UnifiedGreatMod’ workflow can be summarized in the following three phases:

1. definition of the mechanistic-based model and identification of constraint-based model;
2. identification of the reactions encoded in the constraint-based model (i.e. metabolic network) to exploit the harmonization by coordinating the two modelling techniques;
3. simulation the unified system.

Notably, the ‘UnifiedGreatMod’ workflow allows for a flexible architecture that enables users to develop custom pre-processing steps tailored to their specific research needs, provided they adhere to the paradigm established mathematical formalism. The definition of coupling encompasses two steps that enable the complete integration of omics data: the definition of the mechanistic-based model and the identification of the constraint-based model. At these levels, pre-processing modules defined by users can operate within the formal constraints of the FBA and Petri net formalisms, ensuring mathematical consistency while maintaining biological relevance.

#### 2.1.1 Phase 1—Step 1: definition of the mechanistic model

In this first step of the first phase, the user initially draws the mechanistic-based model using the graphical Petri Net (PN) formalism.

In detail, PNs (Marsan *et al.* 1995) and its generalizations, such as the Extended Stochastic PNs (ESPNs) (Pernice *et al.* 2020a), are a high-level mathematical formalism that exploits graphical elements to represent system components and their interactions. ESPN formalism has been extensively used to study biological systems (Castagno *et al.* 2020, Pernice *et al.* 2020b, Pernice *et al.* 2023), thanks to its ability to represent reaction systems in a natural graphical manner and provide qualitative and quantitative information on the system’s behaviour under study.

The basic components are (i) ‘places’, corresponding to state variables of the system (graphically represented as circles) and (ii) ‘transitions’, denoting events or activities (graphically represented as boxes). Places can contain ‘tokens’ drawn as black dots (namely marking of the place), representing the modelled entities of the system. Then, the number of tokens in each place defines the state of a PN, called ‘marking’. Places and transitions are connected by ‘arcs’, which express the relation between states and event occurrences. Moreover, by definition, ESPN can successfully integrate complex velocities functions in a unique model, and combine metabolic and regulatory networks (see section 2.1.4).

An example of ESPN is depicted in the first-top layer of Fig. 1, which is characterized by four places (named ‘P1’, ‘P2’, ‘P3’, and ‘P4’) and seven transitions (named ‘T1’, ‘T2’, ‘$T_{v2,\mathrm{out}}$’, ‘$T_{v2,\mathrm{in}}$’, ‘$T_{v3,\mathrm{out}}$’, ‘$T_{v3,\mathrm{in}}$’, and ‘$T_{v4,\mathrm{out}}$’). For instance, the ‘T1’ transition is connected to two input places ‘P1’ and ‘P4’ and two output places (‘P2’ and ‘P3’). Thus, it can change the marking of the four places through its ‘firing’, i.e. by removing tokens from ‘P1’ and ‘P4’, and adding tokens to ‘P2’ and ‘P3’ with a firing intensity defined by the Mass Action law: $c_{t_{1}}x_{P_{1}}(\nu )x_{P_{4}}(\nu )$. Where $c_{t_{1}}$ is the kinetic constant of the transition and $x_{P_{1}}(\nu ),\;x_{P_{4}}(\nu )$ are the number of tokens at time ν in the places $P_{1}$ and $P_{4}$, respectively.

As shown in Pernice *et al.* (2020a), starting from an ESPN model, it is possible to derive the system of ODEs describing how the number of tokens changes over time in each place, as graphically represented by the red dashed arrow in Fig. 1. For instance, if we consider the first ODE in the rectangle pointed by the red dashed arrow, its solution describes the evolution in the number of tokens in the place ‘P1’ over time.

Since ‘P1’ has only one transition (‘T1’) that can alter its token count, specifically by removing tokens with an intensity defined as described in the previous paragraph, the changes in token dynamics can be explicitly modeled through this equation.

The same for the remaining three places.

For the model construction and automatic derivation of the low-level mathematical processes characterizing the system dynamics, we exploited and appropriately extended ‘GreatMod’, a general modelling framework, to simulate complex biological systems using an intuitive graphical interface. ‘GreatMod’ is composed of three main modules: (i) a Java GUI based on Java Swing Class, called GreatSPN (Amparore *et al.* 2016), which allows the user to draw models using PN formalism and its extensions, (ii) the R library *epimod* (Castagno *et al.* 2020), and (iii) Docker containerization, a lightweight OS-level virtualization. Further details are reported in the Supplementary Material S1.1.4.

Regarding the parametrization of the mechanistic model, various omics data (e.g. transcriptomics, proteomics) can be integrated to help define reaction activities, map cellular processes under specific conditions, and adjust reaction constraints appropriately. Specifically, transcriptomics data can provide information on the initial values of biological entities or rate constants in the mechanistic model. For instance, gene-protein-reaction (GPR) associations can translate transcriptomics or proteomics data into reaction activities within the network (Di Filippo *et al.* 2021, Zampieri *et al.* 2023). Furthermore, omics data can suggest including specific pathways or biological relationships, ensuring the model captures the full range of dynamics in the system under study.

#### 2.1.2 Phase 1—Step 2: identification of the metabolic network

For metabolic network identification, the corresponding model can be encoded in MAT-file (‘.mat’) format as defined by the COBRA Toolbox (Heirendt *et al.* 2017). Then, the constrained-based modelling approach, called FBA, can be used to study metabolic networks by analysing the flow of metabolites.

A linear programming solver is thus used to find the flux distribution $v\in R^{r}$ of all the r reactions that maximize or minimize the objective function $f_{\mathrm{obj}}:R^{r}\to R$ while satisfying two main constraints: (i) the steady-state assumption S·v=0, and (ii) each flux must take value between $v_{i}^{\min}$ and $v_{i}^{\max}$, which are the lower and upper constraints of the i-th flux. Let us denote F, the set of all the fluxes $v_{i},\;i=1,\;\ldots ,\;r$.

To facilitate the integration of a constrained-based model into our framework, we developed the ‘*epimod_FBAfunctions*’ R package. This package offers essential functions to read and modify MAT files, translating metabolic network representations into the ‘GreatMod’ internal format. In particular, it is possible to integrate gene expression data and dietary inputs to set the flux constraints properly or define substrate availability (Cooke *et al.* 2024). Additionally, metabolomics data can play a complementary role by directly influencing reaction bounds, substrate availability, and the initial concentrations of metabolites. We have implemented a strategy to integrate metabolomics and gene expression data. Our computational pipeline can (i) compute differential reaction activities from transcriptomics to predict whether the differential expression of metabolic enzymes directly causes differences in metabolic fluxes and (ii) constrain boundary reactions based on the concentration of metabolites resulting from a given dietary input.

The critical aspect of integrating transcriptomics data lies in selecting the integration method. The package ‘epimod_FBAfunctions’ implements the integration process presented in Kashaf *et al.* (2017). This method evaluates the GPR rules associated with each reaction to derive reaction expression values from gene expression values.

#### 2.1.3 Phase 2—Step 1: identification of the reactions

The first step of the second phase aims to identify a set of boundary reactions, also known as exchange or transport reactions, $F^{\mathrm{shared}}\in F$, so each corresponding flux can be associated with a velocity in the ODEs system, representing the exchange of such metabolites. These reactions define the input and output of metabolites to and from the metabolic network in FBA. These reactions are crucial for accurately modelling the system because they set limits on the availability and removal of metabolites.

First, we need to filter the set of all the fluxes of the FBA model to consider reactions that can be used to define $F^{\mathrm{shared}}$. Specifically, we strongly suggest using only the boundary reactions for two reasons: (i) to maintain consistency with the fundamentals of our paradigm and (ii) not to violate the balance assumption of the FBA model. Our new paradigm has to connect two modelling approaches with their assumptions and rules, simulating two different system layers that share a given number of resources (e.g. metabolites). In particular, the FBA model is constrained by the system’s steady state, which supposes no changes in any metabolite concentration. Therefore, by defining a single ODE that models the concentration over time of a metabolite also described in the FBA model, it is straightforward that the steady-state assumption of the FBA model does not hold anymore. Differently, the boundary reactions represent by definition the connection of the metabolic model with an extracellular environment, delineating in such a manner the perfect bridge between an ODE representing the shared metabolite outside the metabolic model, which acts as its resource by modifying dynamically the reaction constraints, respectively.

Among the boundary reactions, the ones with a greater sensibility to perturbations in their constraints play a central role in connecting the two modelling approaches. Given that the system of ODEs is connected to the FBA model in terms of the fluxes constraints, it is straightforward to consider reactions where small perturbations in the concentration of the shared metabolite affect the FBA solution. Otherwise, the two approaches could be solved independently without dynamic communication, which might improve and change the analysis. Therefore, we suggest focusing the modelling efforts on high-sensitive reaction fluxes to minimize the poor call of such boundaries and reduce the likelihood of errors. We focus on fluxes for which a small perturbation entails a substantial variation of the FBA model outcomes (e.g. the objective function value). Specifically, by exploiting Sobol’s variance-based Sensitivity Analysis (SA) (Saltelli *et al.* 2010), a powerful and widely used method to determine the contribution of input variables to the output variability of a mathematical model, it is possible to detect automatically critical fluxes in genome-scale metabolic, which is particularly useful for understanding how changes in the fluxes constraints influence the objective function. In this context, we developed in ‘epimod_FBAfunctions’ package functionality to perform the SA considering a constrained-based model.

Further details regarding the definition of $F^{\mathrm{shared}}$ and the definition of the SA approach are reported in the Supplementary Material S1.3 and S1.1.3, respectively.

#### 2.1.4 Phase 2—Step 2: harmonization

Once $F^{\mathrm{shared}}$ is defined, the next step is defining how to coordinate the two modelling approaches.

The general idea is that the concentrations obtained from solving the ODEs could be used as flux constraints because they represent the availability of the metabolites in the environment and, therefore, the uptake limit. Usually, the upper constraint is set only if a growth limit has to be defined. Otherwise, it should be infinity (see the Supplementary Material S1.4).

Thus, given an ESPN model, we consider $T_{g}$ the set of all transitions (called general transitions) whose firing intensities are defined as continuous real functions, and not through the Mass Action law. Let us refine $T_{g}$ into two disjoint subsets, $T_{g}^{\mathrm{FBA}}$ and $T_{g}^{\neg \mathrm{FBA}}$, so that (i) $T_{g}^{\mathrm{FBA}}\cup T_{g}^{\neg \mathrm{FBA}}=T_{g}$ and (ii) $t\in T_{g}^{\mathrm{FBA}}$ if only if its intensity is obtained by solving the constrained-based model.

Let us observe that, by definition, the transition intensity has to be a positive number, therefore if reversible reactions are considered in $F^{\mathrm{shared}}$, then they are decoupled into irreversible reaction pairs, defining two different transitions, denoted with _in if the estimated flux is negative and _out if it is positive. Thus, we can infer that


(1) $$\begin{array}{c} \begin{array}{cc} \forall v_{k}\in F^{\mathrm{shared}}\Rightarrow & \exists \;t_{v_{k},in}\;\wedge \;t_{v_{k},\mathrm{out}}\in T_{g}^{\mathrm{FBA}} \end{array}\\ \begin{array}{c} s.t.\;\;f_{t_{v_{k},\mathrm{out}}}(\nu ,\hat{x})=1_{\left(v_{k}\geq 0\right)}v_{k}\;\wedge \\ \;\;f_{t_{v_{k},in}}(\nu ,\hat{x})=-1_{\left(v_{k}\leq 0\right)}v_{k} \end{array} \end{array}$$

where, $f_{t}()$ is the firing intensity associated with the general transition $t\in T_{g}^{FBA}$ depending on the marking of its inputs places $\hat{x}$ at time ν, $t_{v_{k},in/\mathrm{out}}$ the general transition representing the flux $v_{k}$, and $1_{\left(\cdot \right)}$ is the indicator function that returns 1 only if the input condition is satisfied.

As depicted with the green colour in Fig. 1, $F^{\mathrm{shared}}$ is defined by three fluxes $v_{2},v_{3},v_{4}$ associated, respectively, with the boundary reactions $r_{EX\_P_{2}},r_{EX\_P_{3}},r_{EX\_P_{4}}$. Let us observe that these three reactions were selected since they represent the exchange of the metabolites represented by the places $P_{2}$, $P_{3}$, and $P_{4}$, respectively. Therefore, for each reaction, one or two general transitions are defined depending on whether it is a reversible reaction or not. For instance, the reaction $r_{EX\_P_{2}}$ is associated with two general transitions $T_{v_{2},in}$ and $T_{v_{2},\mathrm{out}}$, whose intensity will depend on the sign of $v_{2}$ obtained by solving the FBA.

Let us observe that the constraints of the shared fluxes are defined as functions $v_{k}^{\min}=f_{\mathrm{lower}}(v_{k},\hat{x})$ and $v_{k}^{\max}=f_{\mathrm{upper}}(v_{k},\hat{x})$ (lower and upper bounds, respectively) that depend on the solution of the system of ODEs thought $\hat{x}$, i.e. the input places of the transitions in $T_{g}^{\mathrm{FBA}}$. For instance, they might be defined as a linear transformation of the respective exchanged metabolites concentration (i.e. $x_{J^{-1}(k)}$) when $i\in M^{Ex}$.

#### 2.1.5 Phase 3: simulation

Finally, the simulation technique integrates the solution of the system of ODEs, which is based on the Backward Differentiation Formula method (Burden *et al.* 2016), with the resolution of the Linear Programming Problem (The Backward Differentiation Formula method is implemented using the C++ LSODA library (https://en.smath.com/view/lsoda), while the optimization problem using the *glpk* library (https://www.gnu.org/software/glpk/).

Specifically, this approach relies on a threshold criterion that compares the elements of the ODEs affecting the FBA (i.e. some lower and upper bounds might change) at the two consecutive solution times. Consequently, the FBA is performed with the new constraints solely when notable alterations occur enabling the ODEs to be adjusted to reflect substantial changes in the FBA model dictated by flux constraints and input specifications. This improves the model’s efficiency by avoiding superfluous computations. Therefore, the solution of the model emerges from two closely linked resolutions of the ODEs and FBA (we refer for formal details to the Supplementary Material S1.5 and to Supplementary Material S1.4 for the performance analysis).

An example of the final system of ODEs is depicted in Fig. 1, in which both the components of the mechanistic-based (in red) and constrained-based (in green) models are present.

## 3 Results

The use of deep sequencing technology to analyse microbiome profiles across different tissues has prompted the microbiology community to expand their research objectives to include the functional aspects of microorganisms integrating heterogeneous data. For this reason, we propose two applications of ‘UnifiedGreatMod’ in this context.

The first application is related to the carbon catabolite repression phenomenon exhibited by *E. coli* (Ammar *et al.* 2018), with all details provided in the Supplementary Materials.

The second application, described hereafter, investigates the interaction between *Clostridioides difficile* and intestinal epithelial cells (IECs).

### Definition of mechanistic and constraint-based models

*Clostridioides difficile* is a gram-positive bacteria that causes infections typically affecting older adults in hospitals or long-term care. Symptoms of the clinical disease can range from diarrhoea to life-threatening damage to the colon. During infection, *C. difficile* colonizes the large intestine and causes toxin-mediated disruption of the intestinal epithelial barrier function, which results in increased intestinal permeability and triggers haemorrhage (Kouhsari *et al.* 2018). Recently, evidence has shown that chronic *C. difficile* infection contributes to colorectal tumorigenesis (Drewes *et al.* 2022). To model the interaction between *C. difficile* and IECs during infection, six interacting biological domains have been considered: (i) *C. difficile* ‘metabolic network’ incorporates all metabolic reactions of the *C. difficile*; (ii) *C. difficile* ‘cell dynamics’ models the proliferation response of a bacteria population upon treatment with antimicrobial therapy; (iii) ‘metronidazole action’ describes the mechanism of action of a drug driving an imbalance between oxidative and antioxidative processes, inducing oxidative stress and cell death; (iv) ‘intestinal lumen’ models the toxin-mediated inflammation and the dietary intake of nutrients; (v) ‘intestinal epithelial cells’ describes the dynamics of the IEC and the absorption of nutrients, and (vi) ‘blood vessels’ describes the transepithelial amino acids transported into the circulation following protein digestion and absorption. The detailed biological description of all modules is provided in Supplementary Material S3.1. The model’s parameterization is primarily based on values extracted from the literature, except for ‘Death4Treat’, ‘Detox’, and ‘IECsDeath’. These parameters are explored using Partial Rank Correlation Coefficients (Fornari *et al.* 2015) to observe changes in the overall model dynamics (see Supplementary Fig. S5). Further details are available in Supplementary Material S1.2.1. Additionally, Supplementary Material S2 provides a detailed example of how parameters can be derived from transcriptomic data.

### Model component identification and harmonization

Harmonizing the low-level ODEs and the low-level metabolic model (exchanges between the external environment and the metabolic model) involves identifying the critical fluxes through Sobol’s variance-based SA (Saltelli *et al.* 2010). The SA method indicates which transitions in our model have the highest influence on the output.

The process of selection relies on the identification of the metabolic model’s most sensitive exchange reactions (see the Supplementary Material S1.1.3). We extrapolated sensitivity coefficients for exchange reactions and concentrated on the most sensitive reactions. This approach minimizes the impact of poor boundary selection. A filtering threshold was applied, where reactions associated with sensitivity coefficients below 0.01 (this threshold was arbitrary) were considered negligible. The final selection included ‘EX_leu_L_e’ and ‘EX_trp_L_e’, which are the most sensitive reactions that are also aligned with the biological question of the model.

To better capture all dynamics biologically related to the antibiotic-resistant mechanism, (Karasawa *et al.* 1995, Knippel *et al.* 2020), we also consider the transitions ‘EX_pro_L_e’, ‘EX_ile_L_e’, ‘EX_val_L_e’, ‘EX_cys_L_e’, and ‘sink_pheme_c’. These reactions are split into ‘in’ and ‘out’ components to retain direction information.

For a detailed formal description of the harmonization process, we refer to the Supplementary Material S1.2.

### Modelling antibiotic therapy across experimental settings and conditions

**Figure 2.**
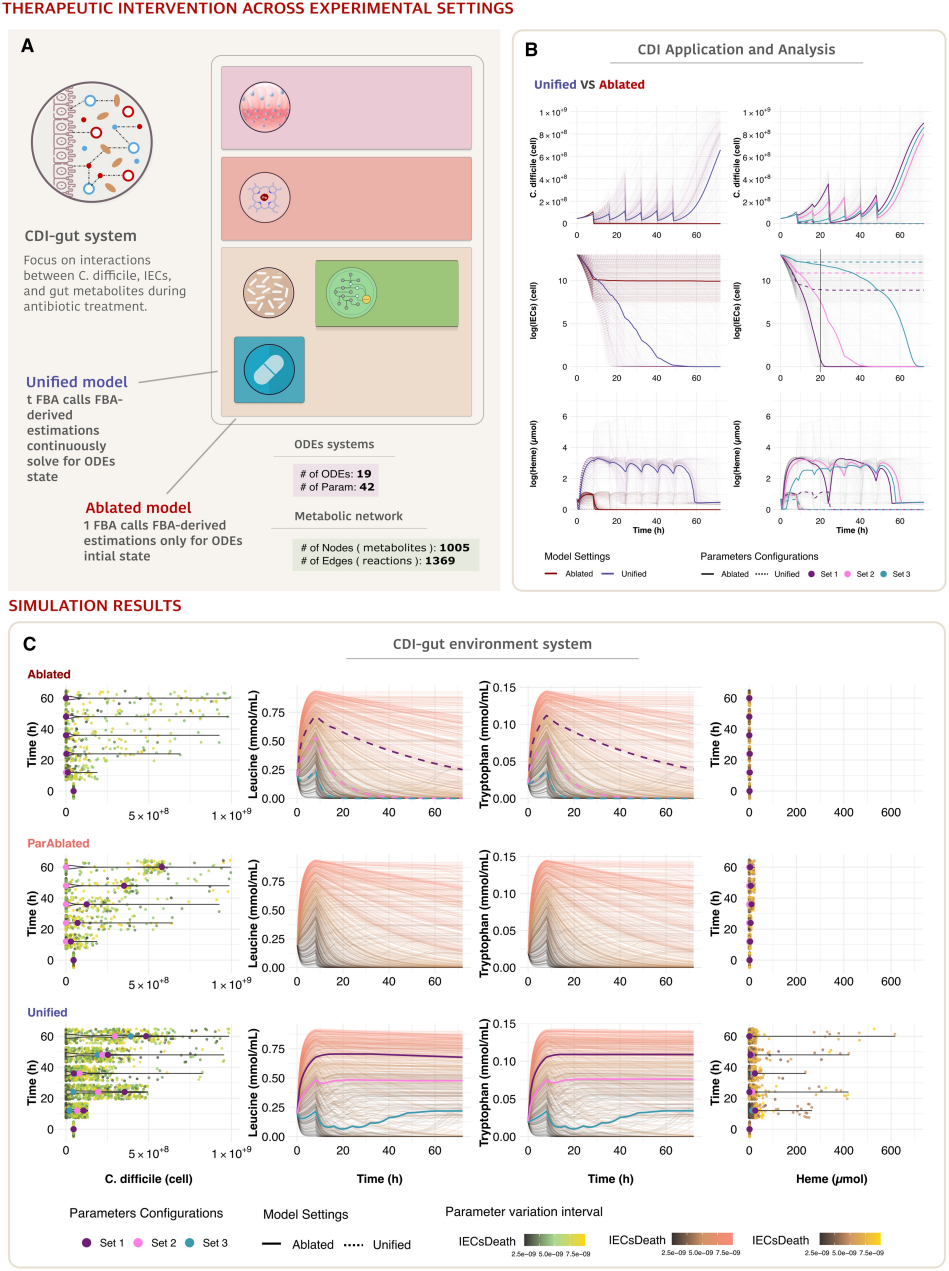
Analysis of *C. difficile* infection dynamics across different modelling settings. (A) Schematic representation of the CDI-gut system model architecture: illustration of interactions between *C. difficile*, intestinal epithelial cells (IECs), and gut metabolites during antibiotic treatment. (B) Comparative analysis of model behaviour: The left panels present the median trajectories of the Ablated model compared to the Unified model, while the right panels illustrate the same variables, colour-coded by three different parameter configuration sets. These visualizations highlight distinct behavioural patterns in bacterial population dynamics, IEC survival, and metabolic adaptation. (C) CDI-gut environment system analysis across experimental settings: violin plots with overlaid trajectories showing *C. difficile* population, leucine and tryptophan concentrations, and haem levels, demonstrating how different modelling approaches capture system heterogeneity and metabolic-dynamic coupling.

The coupled model runs simulations to predict the system behaviour under different experimental settings and conditions. To assess the significance of ‘UnifiedGreatMod’, we proposed three experiment settings. In the first, called ‘ablation experiment’, the dynamics of the metabolic environment are computed by FBA only at the beginning of the simulation. In the second experiment, named ‘partial-ablation experiment’, the FBA is computed when an external stimulus occurs, i.e. at any drug injection. In the last experiment, namely ‘unified experiment’, the FBA model is continuously solved for the state of the ODEs system (Fig. 2A).

Moreover, system dynamics are also responsive according to different systems conditions, where a condition is represented by given systems parameters configuration selected after a calibration step aimed to (i) minimize the IECs damage, (ii) keep the drug concentration closed to the minimum inhibitory concentration value, and (iii) minimize the number of *C. difficile* cells without complete eradication.

In the unified setting, in a condition of limited drug efficacy, the successful colonization by *C. difficile* leads to an increment in the number of *C. difficile* cells after the completion of therapy injections. Consequently, IECs die, the gut lining is damaged, and, following extravasation, red blood cells undergo lysis, which increases heame concentration in the gut micro-environment Fig. 2B (left panels). Furthermore, only the Unified experiment reveals the presence of a haeme-mediated spectrum of antibiotic-resistant strains, ranging from fully susceptible to partially susceptible and fully resistant populations. The model results illustrate how a gradient of conditions fosters diverse antibiotic resistance types (Fig. 2B).

Three representative parameter configurations were identified based on median trajectories of ${\text{IECs}}_{t=20}$. These configurations (Sets 1–3; parameter values in Supplementary Table S3) demonstrate distinct infection phenotypes: Set 1 exhibits the highest bacterial growth and IEC death rate, indicating strong antibiotic resistance, Set 2 shows intermediate behaviour, while Set 3 displays controlled bacterial growth and enhanced IEC survival suggesting higher antibiotic susceptibility (Fig. 2B, right panels).

The drug therapy is administered as injections at specific time intervals (8, 16, 24, 32, 40, and 48 h). In the Unified scenario, a decrease in *C. difficile* count is observed following each drug injection. Meanwhile, the number of IECs declines due to the lethal effects of the infection, leading to an increase in intracellular haem concentration. A comprehensive overview of the model results across all locations is provided in Supplementary Fig. S4.

The comparison across experimental settings reveals different trajectories in the evolution of the system (Fig. 2C). For instance, in all three settings, the number of IECs changes linearly with the IECsDeath rate, representing the degree of infection virulence. However, the impact of the Unified model becomes particularly evident when analysing cell numbers: as cell death increases, the decline in IEC count is significantly more pronounced in the Unified model compared to the Ablated settings. Additionally, distinct behavioural differences emerge in each experimental setting when examining the trajectories of leucine, tryptophan, and intracellular haem (Fig. 2C).

## 4 Discussion

Computational modelling is becoming increasingly crucial to formulate new hypotheses and provide plausible explanations of how a real system works. *In-silico* models may help uncover how system components interact with each other and how they are related to wider biological effects. However, new computational theoretical and practical achievements are needed to model how cell dynamics are intertwined with the mechanics of cell signalling, metabolism, and the influence of extracellular factors.

Here, we propose ‘UnifiedGreatMod’ based on open-source and general framework to integrate formal concepts and different solution techniques and offer the possibility of relating multi-level dynamics. ‘UnifiedGreatMod’ exploits a single easy-to-use graphical meta-formalism ensuring result reproducibility. The most innovative aspect of ‘UnifiedGreatMod’ is the possibility to continuously compute the evolution of the systems considering, at the same time, all the reactions involved in the metabolism, and changes in the micro-environment. Due to its clinical importance as a nosocomial disease and an emerging tumorigenic pathogen, ‘UnifiedGreatMod’ was exploited to model *C. difficile* infection in the human gut as a case study.

In the gut, the growth medium composition significantly affects *C. difficile*’s toxin production. These toxins compromise the epithelial cell barrier, causing apoptosis in IECs. Toxin synthesis regulation is a complex bacterial response to specific nutrient availability during infection. *Clostridioides difficile* virulence is tightly linked to nutrient availability; when nutrients are limited, the bacteria produce toxins. These toxins release host-derived nutrients through cytotoxic activity. When IECs die, they release their intracellular contents, including nutrients such as amino acids, into the gut environment. These nutrients then become available for the bacteria.

Leucine is a key metabolite of *C. difficile* Stickland fermentation, i.e. it can be used to generate ATP through its paired oxidation and reduction with other amino acids. It has been shown that leucine is one of the main energy sources during bacterial exponential growth and that its pools are depleted before the onset of the stationary phase (Hofmann *et al.* 2018). The role of tryptophan in the growth of *C. difficile* is not explicitly defined in the literature. However, *C. difficile* cannot synthesize tryptophan, as suggested by Neumann-Schaal *et al.* (2019). It would need to obtain this amino acid from the environment, which could influence its growth dynamics.

The comparison among ablated, partially ablated, and unified experiments demonstrated that simulations depict the connection between nutrient availability in the gut and bacterial growth state during the disease. In all scenarios, tryptophan and leucine dynamics are associated with the model parameter representing the cytotoxic capacity of the bacterium upon IECs. This parameter reflects the degree of toxic gene regulation by nutrient-sensing transcriptional repressors, which are depressed with low levels of environmental nutrients (Theriot and Fletcher 2019).

Nevertheless, by dynamically computing the FBA throughout the entire simulation, we can uncover mechanisms of antibiotic resistance. This mechanism may promote chronic infection by *C. difficile*, even when metronidazole treatment is ongoing.

Antibiotic injections reduce the number of bacteria at drug administration. Ablation experiments indicate that the model shows substantial variations in haem content at comparable equivalent levels of cellular damage. In contrast, the latest experiments better simulate the system’s behaviour by representing a greater richness, showing *C. difficile* acquisition of haem and the ability to withstand oxidative stress are crucial factors of antibiotic resistance. Intermediate susceptibility states are visible from the *C. difficile* dynamics. Highly susceptible bacteria are cleared, preserving IEC barrier integrity and reducing nutrient leakage, while highly resistant bacteria kill IECs, saturating the environment with nutrients. The unified model fanned out trajectories within the intermediate antibiotic resistance category, reflecting real bacterial populations, which typically display a range of antibiotic susceptibilities rather than discrete categories. This gradient allows the model to show how intermediate types are selected and how to establish antibiotic resistance. Additionally, the two FBA integrated experiments capture the dynamic consumption of metabolites due to oscillations in the bacterial population with varying detail.

In conclusion, these results indicate that this new paradigm, harmonizing dynamic and metabolic models, can significantly enhance personalized healthcare in the context of dynamic treatment and patient monitoring. ODEs allow for the precise modelling of temporal changes in biological systems, capturing the dynamic behaviour of disease progression and treatment response. Meanwhile, FBA enables the optimization of metabolic pathways by analysing the flow of metabolites through networks, ensuring that cellular processes are efficiently managed. By integrating these two powerful approaches, healthcare providers can develop highly tailored therapeutic strategies that are continuously adjusted based on real-time patient data. In the current implementation of UnifiedGreatMod, users must define and link the FBA problem to the ODE system at the beginning of the simulation. While this setup allows users to manually modify the code to implement alternative strategies beyond the classical FBA approach, future updates could improve usability by incorporating modular new functionalities. These enhancements will enable users to choose the integration module suitable for the aim of the study. In addition, the user could also define new integration methods to combine bio-omics data without needing significant manual modifications.

## Supplementary Material

btaf103_Supplementary_Data

## Data Availability

GreatMod https://qbioturin.github.io/epimod/, epimod_FBAfunctions https://github.com/qBioTurin/epimod_FBAfunctions, first case study *E. coli*  https://github.com/qBioTurin/Ec_coli_modelling, second case study *C. difficile*  https://github.com/qBioTurin/EpiCell_CDifficile.

## References

[btaf103-B1] Ammar EM , Wang X, Rao CV et al Regulation of metabolism in *Escherichia coli* during growth on mixtures of the non-glucose sugars: arabinose, lactose, and xylose. Sci Rep 2018;8:609. 29330542 10.1038/s41598-017-18704-0 PMC5766520

[btaf103-B2] Amparore E, Balbo G, Beccuti M et al *30 years of greatSPN*, London: Springer Series in Realibility Engineering, 2016, 227–54.

[btaf103-B3] Ben Guebila M , Thiele I. Dynamic flux balance analysis of whole-body metabolism for type 1 diabetes. Nat Comput Sci 2021;1:348–61. 38217214 10.1038/s43588-021-00074-3

[btaf103-B4] Bordbar A , Monk JM, King ZA et al Constraint-based models predict metabolic and associated cellular functions. Nat Rev Genet 2014;15:107–20. 24430943 10.1038/nrg3643

[btaf103-B5] Burden RL , Feires JD, Burden AM. *Numerical Analysis*, 10th edn. Boston: Cengage Learning, 2016.

[btaf103-B6] Büchel F , Rodriguez N, Swainston N et al Path2models: large-scale generation of computational models from biochemical pathway maps. BMC Syst Biol 2013;7:116. 24180668 10.1186/1752-0509-7-116 PMC4228421

[btaf103-B7] Castagno P , Pernice S, Ghetti G et al A computational framework for modeling and studying pertussis epidemiology and vaccination. BMC Bioinformatics 2020;21:344. 32938370 10.1186/s12859-020-03648-6 PMC7492136

[btaf103-B8] Cooke J , Delmas M, Wieder C et al Genome scale metabolic network modelling for metabolic profile predictions. PLoS Comput Biol 2024;20:e1011381. 38386685 10.1371/journal.pcbi.1011381 PMC10914266

[btaf103-B9] Damiani C , Maspero D, Di Filippo M et al Integration of single-cell rna-seq data into population models to characterize cancer metabolism. PLoS Comput Biol 2019;15:e1006733. 30818329 10.1371/journal.pcbi.1006733 PMC6413955

[btaf103-B10] Di Filippo M , Pescini D, Galuzzi BG et al.. *Integrate: Model-based multi-omics data integration to characterize multi-level metabolic regulation*, PLoS Comput Biol 2021;10:e1009337. 10.1371/journal.pcbi.1009337 PMC8853556 35130273

[btaf103-B11] Drewes JL , Chen J, Markham NO et al Human colon cancer–derived *Clostridioides difficile* strains drive colonic tumorigenesis in mice. Cancer Discov 2022;12:1873–85. 35678528 10.1158/2159-8290.CD-21-1273 PMC9357196

[btaf103-B12] Feist AM , Herrgård MJ, Thiele I et al Reconstruction of biochemical networks in microorganisms. Nat Rev Microbiol 2009;7:129–43. 19116616 10.1038/nrmicro1949 PMC3119670

[btaf103-B13] Fornari C , Balbo G, Halawani SM et al A versatile mathematical work-flow to explore how cancer stem cell fate influences tumor progression. BMC Syst Biol 2015;9. 10.1186/1752-0509-9-S3-S1 PMC4464028 26050594

[btaf103-B14] Galuzzi BG , Vanoni M, Damiani C et al Combining denoising of RNA-seq data and flux balance analysis for cluster analysis of single cells. BMC Bioinformatics 2022;23:445. 36284276 10.1186/s12859-022-04967-6 PMC9597960

[btaf103-B15] Heirendt L , Arreckx S, Pfau T et al Creation and analysis of biochemical constraint-based models: the cobra toolbox v3.0. Nature Protocols 2017;14:639–702. 10.1038/s41596-018-0098-2 PMC6635304 30787451

[btaf103-B16] Hofmann JD , Otto A, Berges M et al Metabolic reprogramming of *Clostridioides difficile* during the stationary phase with the induction of toxin production. Front Microbiol 2018;9:1970–17. 30186274 10.3389/fmicb.2018.01970 PMC6110889

[btaf103-B17] Karasawa T , Ikoma S, Yamakawa K et al A defined growth medium for *Clostridium difficile*. Microbiology (Reading) 1995;141(Pt 2):371–5. 7704267 10.1099/13500872-141-2-371

[btaf103-B18] Kashaf SS , Angione C, Lió P et al Making life difficult for clostridium difficile: augmenting the pathogen’s metabolic model with transcriptomic and codon usage data for better therapeutic target characterization. BMC Syst Biol 2017;11:25. 28209199 10.1186/s12918-017-0395-3 PMC5314682

[btaf103-B19] Kendall BE , Briggs CJ, Murdoch WW et al Why do populations cycle? A synthesis of statistical and mechanistic modeling approaches. Ecology 1999;80:1789–805.

[btaf103-B20] Knippel RJ , Wexler AG, Miller JM et al *Clostridioides difficile* senses and hijacks host heme for incorporation into an oxidative stress defense system. Cell Host Microbe 2020;28:411–21.e6. 32526159 10.1016/j.chom.2020.05.015 PMC7486240

[btaf103-B21] Kouhsari E , Abbasian S, Sedighi M et al *Clostridium difficile* infection: a review. Rev Res Med Microbiol 2018;29:103–9.

[btaf103-B22] Machado D , Andrejev S, Tramontano M et al Fast automated reconstruction of genome-scale metabolic models for microbial species and communities. Nucleic Acids Res 2018;46:7542–53. 30192979 10.1093/nar/gky537 PMC6125623

[btaf103-B23] Machado D , Herrgård M. Systematic evaluation of methods for integration of transcriptomic data into constraint-based models of metabolism. PLoS Comput Biol 2014;10:e1003580. 24762745 10.1371/journal.pcbi.1003580 PMC3998872

[btaf103-B24] Magnúsdóttir S , Heinken A, Kutt L et al Generation of genome-scale metabolic reconstructions for 773 members of the human gut microbiota. Nat Biotechnol 2017;35:81–9. 27893703 10.1038/nbt.3703

[btaf103-B25] Mahadevan R , Edwards JS, Doyle FJ et al Dynamic flux balance analysis of diauxic growth in *Escherichia coli*. Biophys J 2002;83:1331–40. 12202358 10.1016/S0006-3495(02)73903-9 PMC1302231

[btaf103-B26] Maranas D , Zomorrodi A. Optimization Methods in Metabolic Networks. New York, USA: Wiley, 2016.

[btaf103-B27] Marsan A et al Modelling with Generalized Stochastic Petri Nets. New York, NY: J. Wiley, 1995.

[btaf103-B28] Neumann-Schaal M , Jahn D, Schmidt-Hohagen K et al Metabolism the difficile way: the key to the success of the pathogen *Clostridioides difficile*. Front Microbiol 2019;10:219. 30828322 10.3389/fmicb.2019.00219 PMC6384274

[btaf103-B29] Noronha A , Modamio J, Jarosz Y et al The virtual metabolic human database: integrating human and gut microbiome metabolism with nutrition and disease. Nucleic Acids Res 2019;47:D614–D24. 30371894 10.1093/nar/gky992 PMC6323901

[btaf103-B30] Norsigian CJ , Pusarla N, McConn JL et al Bigg models 2020: multi-strain genome-scale models and expansion across the phylogenetic tree. Nucleic Acids Res 2020;48:D402–D6. 31696234 10.1093/nar/gkz1054 PMC7145653

[btaf103-B31] Opdam S , Richelle A, Kellman B et al A systematic evaluation of methods for tailoring genome-scale metabolic models. Cell Syst 2017;4:318–29.e6. 28215528 10.1016/j.cels.2017.01.010 PMC5526624

[btaf103-B32] Palsson B. *Systems Biology: Simulation of Dynamic Network States*. Cambridge, UK: Cambridge Press, 2011.

[btaf103-B33] Pernice S , Follia L, Balbo G et al Integrating petri nets and flux balance methods in computational biology models: a methodological and computational practice. Fundamenta Informaticae 2020a;171:367–92.

[btaf103-B34] Pernice S , Follia L, Maglione A et al Computational modeling of the immune response in multiple sclerosis using epimod framework. BMC Bioinformatics 2020b;21:550. 33308135 10.1186/s12859-020-03823-9 PMC7734848

[btaf103-B35] Pernice S , Maglione A, Tortarolo D et al A new computational workflow to guide personalized drug therapy. J Biomed Inform 2023;148:104546. 37984546 10.1016/j.jbi.2023.104546

[btaf103-B36] Saltelli A , Annoni P, Azzini I et al Variance based sensitivity analysis of model output. design and estimator for the total sensitivity index. Comput Phys Commun 2010;181:259–70.

[btaf103-B37] Theriot CM , Fletcher JR. Human fecal metabolomic profiling could inform *Clostridioides difficile* infection diagnosis and treatment. J Clin Invest 2019;129:3539–41. 31403467 10.1172/JCI130008 PMC6715351

[btaf103-B38] Yang L , Ebrahim A, Lloyd CJ et al Dynamicme: dynamic simulation and refinement of integrated models of metabolism and protein expression. BMC Syst Biol 2019;13:2. 30626386 10.1186/s12918-018-0675-6 PMC6327497

[btaf103-B39] Zampieri G , Campanaro S, Angione C et al Metatranscriptomics-guided genome-scale metabolic modeling of microbial communities. Cell Rep Methods 2023;3:100383. 36814842 10.1016/j.crmeth.2022.100383 PMC9939383

