# Supplementary material for: UnifiedGreatMod: a new holistic modelling paradigm for studying biological systems on a complete and harmonious scale
Source: Bioinformatics. 2025 Mar 12;41(3):btaf103. doi: 10.1093/bioinformatics/btaf103 (PMC11932724; doi:10.1093/bioinformatics/btaf103)
Supplement: btaf103_Supplementary_Data [file btaf103_supplementary_data.pdf]

Supplementary Material

Table of contents:

- **1. Theoretical Background**
  - Petri Net formalism and its extensions
  - Flux Balance Analysis
  - Global Sensitivity Analysis
  - The GreatMod framework
- **2. The Harmonisation Paradigm**
  - Harmonisation definition
  - Reaction identification
  - Constraints definition
  - Synchronisation
- **3. E. coli unified model**
  - Overview of the modelling approach.
  - Methodology
  - Results
- **4. Modelling host and drug responses to Clostridium difficile infection**
  - Metabolic Basis of C. difficile pathogenesis
  - Physiological alterations during antibiotic perturbation
  - Drug detoxification response modulated by micronutrients
  - *UnifiedGreatMod* application: C. difficile infection
  - Tables
  - Figures
- **5. Performance valuation**
  - *Escherichia coli* unified model
  - *Clostridium difficile* infection

Theoretical Background

Petri Net formalism and its extensions

Petri Nets (PNs) (Marsan *et al.*, 1995) and their extensions are widely recognised to be a powerful tool for modelling and studying complex systems thanks to their ability to represent systems in a natural graphical manner and of allowing the computation of qualitative and quantitative information about the behaviour of these systems. In the literature, several generalisations of this formalism are presented to expand the possibility of modelling complex systems of different kinds (Herajy *et al.*, 2018; Pernice *et al.*, 2019). In this work, we will exploit the Extended Stochastic PN (ESPN), a PN generalisation allowing the definition of complex rate functions.

More specifically, ESPNs are bipartite directed graphs with two types of nodes: *places*, which correspond to state variables of the system, graphically represented as circles, and *transitions*, which correspond to the events that can generate a state change and graphically are represented as boxes. Places can contain *tokens* drawn as black dots, representing the modelled entities of the system. Then, the number of tokens in each place defines the state of an ESPN, called *marking*.

Nodes of different types are connected by *arcs*, which express the relation between states and event occurrences. A specific multiplicity is associated with each arc, and it describes the number of tokens removed from (or added to) the corresponding place upon the firing of the transition to which the arc is connected. Graphically, it is written beside the arc, but the default value of one is omitted. Functions I and O describe transitions' input and output arcs, respectively. For convenience, these can be represented by $n_t \times n_p$ matrices of natural numbers, where n_t is the size of the transitions set T, and n_p of the set of places P. The matrix $L = O - I$ is called the *incidence matrix* (Colom and Silva, 2006), representing the transitions' overall effect.

Finally, the evolution of the system is given by the firing of enabled transitions, where a transition is enabled if each input place contains several tokens greater than or equal to a given threshold defined by the multiplicity of the corresponding input arc. Enabled transitions may *fire*, removing a fixed number of tokens from its input places and adding a fixed number of tokens into its output places (according to the multiplicity of its input/output arcs).

Finally, each transition is associated with a specific intensity, representing the parameter of the exponential distribution that characterises its firing time. In this context, to easily model events with velocities defined by different and complex functions, the transitions are split into two subsets: T_{ma} and T_g. The former subset contains all transitions that fire with a velocity expressed in Mass Action (MA) law (Voit *et al.*, 2015). The latter includes all transitions whose random firing times are defined as continuous

real functions. Hence, we will refer to the transitions belonging to T_{ma} as standard transitions and as general transitions those in T_g.

Let define $^\bullet\mathbf{t}$ the subset of P containing the input places to transition t, and $\hat{x} = x(\nu)_{|^\bullet\mathbf{t}}$ as the subset of the marking $x(\nu)$ concerning just the input places to transition t. Thus, given a transition $t \in T = T_{ma} \cup T_g$ at the time ν, it will move tokens in state $x_i(\nu)$ with speed defined as follows: (i) $\phi(t_i, \hat{x}) = \lambda(t_i) \prod_k \hat{x}_k^{I(p_k,t_i)}$ iff $t \in T_{ma}$; (ii) $f_t(\nu, \hat{x})$ iff $t \in T_g$. Thus, the instantaneous changes of tokens $x_i(\nu)$ in the i-th place at time ν, is modelled by the following ODE:

$$dx_i(\nu) = \sum_{j=1}^{n_{T_{ma}}} \phi(t_j^*, \hat{x}) L[p_i, t_j^*] d\nu + \sum_{j=1}^{n_{T_g}} f_{\bar{t}_j}(\nu, \hat{x}) L[p_i, \bar{t}_j] d\nu \quad \forall i \in \{1, \ldots, n_P\}. \tag{S1}$$

ESPN formal definition.

Definition S1.1 (Extended Stochastic Petri Net)**.** An Extended Stochastic Petri Net (ESPN) system is a tuple $(P, T, I, O, \mathbf{m}_0, \lambda, \Lambda)$, where:

- $P = \{p_i\}$ is a finite and non empty set of *places*, with $i = 0, \ldots, n_p$, where n_p is the number of places.
- $T = T_{ma} \cup T_g$ is a finite, non-empty set of transitions, with $T_{ma} \cap T_g = \emptyset$. $T_{ma} = \{t_i^*\}_{1 \le i \le n_{T_{ma}}}$ is the set of the $n_{T_{ma}}$ transitions whose speeds follow the MA law. $T_g = \{\bar{t}_i\}_{1 \le i \le n_{T_g}}$ is the set of the n_{T_g} transitions whose speeds are defined as continuous functions.
- $I, O : P \times T \to \mathbb{N}$ are the *input*, *output*, that defines the net's arcs and specifies their multiplicities.
- $\mathbf{m}_0 : P \to \mathbb{N}$ is a multiset on P representing the *initial marking* of the net.
- $\lambda : T_{ma} \to \mathbb{R}$ gives the firing intensity of the transitions.
- $\Lambda = \{f_1, \ldots, f_h\}$ is the firing intensity set grouping the functions characterising the general transitions in T_g, with $h \le n_{T_g}$, $h \in \mathbb{N}$. In particular, a function f depends only on the marking of the input places of the respective transition t ($|^\bullet\mathbf{t}|$) and on time $\nu \in \mathbb{R}^+$, i.e.,
$$f : \mathbb{R}^{|^\bullet\mathbf{t}|} \times \mathbb{R}^+ \longrightarrow \mathbb{R}.$$

For instance the function f_1 might represent a Michaelis Menten kinetic and f_2 an Hill kinetic.

Flux Balance analysis

Flux Balance Analysis (FBA) is a computational approach used in Systems Biology to study the metabolism of biological systems, particularly in microorganisms like bacteria and yeast (Papoutsakis, 2000; Watson, 1984). FBA is a powerful computational tool for predicting cellular metabolism by formulating it as a linear optimisation problem, where the goal is to find the optimal distribution of metabolic fluxes within a cell to achieve a specific biological objective, by assuming the steady state of the system.
The first works ((Papoutsakis, 2000; Watson, 1984)) regarding the FBA date back to the early 1980s and showed the possibility of deriving from a system of metabolic reactions the stoichiometric equations describing the relations among different products and biomass and how to exploit linear programming for deriving the fluxes of such relations. FBA models have wide applications for simulating genome-scale reconstructions of metabolic networks. These networks are distinguished by a multitude of reactions and metabolites. The model operates under the assumption of a system-wide steady state. In particular, the flux distribution within the model is estimated by setting lower and upper boundaries for each flux. This function acts as a surrogate, representing the most plausible physiological state among all potential states of the system.

From a mathematical point of view, cellular metabolism is represented as a stoichiometric matrix that describes the relationships between different metabolites and reactions in the network. Each row of the matrix corresponds to a metabolite, and each column corresponds to a reaction. Fluxes represent the rate of flow of metabolites through each reaction in the network. FBA assumes that the cellular system is at a steady state, meaning that the concentrations of metabolites do not change over time, supposing that there is no net surplus or deficit of any metabolite. Mathematically the FBA modelling m metabolites and r reactions can be translated as a linear programming problem (LPP) as follows:

$$\begin{aligned} \text{Maximise} \quad & f(v) \\ \text{Subject to} \quad & S \cdot v = 0, \\ & v_i^{min} \le v_i \le v_i^{max}, \quad \forall i = 1, \ldots, r, \\ & v_i \in \mathbb{R} \end{aligned} \tag{S2}$$

with $v \in \mathbb{R}^r$ is the flux vector describing the activity of all the r reactions, $S \in \mathbb{R}^{r \times m}$ is the stoichiometric matrix , $f : \mathbb{R}^m \to \mathbb{R}$ is the objective function to maximise, $v_i^{min} \in (-\infty, 0]$ and $v_i^{max} \in [0, \infty)$ are the constraints of the i-th flux.

Constraints are applied to limit the allowable flux through each reaction, often based on experimentally determined rates or other biological constraints, and they are set depending on the type of the reaction ((Hao *et al.*, 2010)): *exchanges* (uptake or secretion of metabolites into or out of the system), *transporters* (movement of metabolites across cellular membranes or compartments within the cell), *internals* (biochemical transformations that occur within the cell), and *demands/sinks* (need for specific metabolites

within the system, or removal of certain metabolites without specifying a source).
In particular, these constraints are set depending on the type of the reaction. Typically metabolic models might be defined by more than one compartment (differential localisation of biochemical reactions within the cell), and so different types of reactions such as *exchanges*, *transporters*, *internals*, and *demands/sinks* can be defined depending on how they communicate within or among the compartments. The objective function is defined as a function $f : E \longrightarrow \mathbb{R}$ where E represents the flux vector satisfying (i) the mass balance equation, and (ii) the constraints (both defined in Eq.s S2).

Exchanges and *demands/sinks* reactions define the different types of predefined *boundary reactions* because they define the limits or boundaries of the metabolic system being modelled. The term *boundary reactions* refers to reactions that involve metabolites entering or leaving the system, however, *exchanges* are usually connected to the extracellular compartment while *demands/sinks*, on the other hand, are typically connected to intracellular compartments.
Exchange reactions represent the uptake or secretion of metabolites between the cell and its surrounding environment. If an exchange reaction is only an uptake (import) reaction, then $v_i^{min} = 0$ and $v_i^{max} = +\infty$. If it is only a secretion (export) reaction, then $v_i^{min} = -\infty$ and $v_i^{max} = 0$. If the exchange reaction can function in both directions (import and export), then $v_i^{min} = -\infty$ $v_i^{max} = +\infty$. For a demand reaction (which consumes a metabolite), $v_i^{min} = 0$ and $v_i^{max} = +\infty$. For a sink reaction (which can either consume or produce a metabolite) $v_i^{min} = -\infty$ and $v_i^{max} = +\infty$.

Conversely, *transporters* and *internals* are types of predefined *core reactions* because they refer to reactions that involve metabolites entering or leaving the system's compartments. *Transporters* represent the transfer of metabolites between two different compartments within the cell. The directionality and limits of these reactions can vary depending on the specific biological system being modelled. *Internal* reactions represent the conversion of one substance into another within the same compartment. Typically, for irreversible internal reactions, $v_i^{min} = 0$ and $v_i^{max} = +\infty$. For reversible internal reactions, $v_i^{min} = -\infty$ and $v_i^{max} = +\infty$.

Global Sensitivity Analysis

FBA is a potent computational method for analysing metabolite flow through a metabolic network. However, the accuracy of FBA predictions is strongly influenced by the choice of flux boundaries, which define the feasible solution space that satisfies the system's constraints.
Inaccurate flux boundary selection can lead to potential errors or unrealistic predictions that do not accurately represent the system's actual behaviour. This issue is particularly critical when choosing flux boundaries for reactions that introduce nutrients into the system, as these boundaries play a crucial role in ensuring biologically relevant and accurate predictions.
To mitigate the risk of such errors, we propose focusing modelling efforts on high-sensitive parameters. These are parameters where minor perturbations can lead to significant variations in model outcomes. By concentrating on these parameters, we can minimise the impact of poor boundary selection and reduce the likelihood of errors.
By integrating global Sensitivity Analysis with FBA, we can enhance the reliability of metabolic models and provide more accurate predictions of metabolite flow through the network.

SA formal definition.

In our study, we utilised Global SA as a computational strategy to examine the impact of various input uncertainties on the output of the FBA model. This approach has been widely used in previous studies to examine complex models, typically presenting sensitivity coefficients for model inputs in sorted order (Qian and Mahdi, 2020).
Our primary objective was to investigate how uncertainties in reaction boundary values influence the objective function of the FBA model. This function signifies the fluxes anticipated to affect the optimal cellular growth rate, which is characterised as the maximisation of biomass production.
We considered scenarios where the indicative value of each parameter was absent. It is a common practice in genome-wide modelling to assign a negative sign to intake fluxes, indicating a direction from outside to inside. To quantify the impact of parameter perturbation corresponding to the lowest boundary, we performed a variance decomposition in the domain $[-10, 0]$ (mmol/gDW/h). We evaluated uncertainties by confining constraints within this interval, based on practical considerations and biological relevance. This interval has been shown to yield biologically relevant results and allows for a broad range of possible flux values (Lularevic *et al.*, 2019).
Also, this interval disposition is a practical method to model nutrient influx boundaries. This approach is designed to establish boundaries that are low enough to accommodate internal reactions and high enough to include the stoichiometric coefficients of reactions involving these nutrients. The aim is to prevent internal boundaries from becoming limiting and to globally sample all possible ratios between intake fluxes by varying all parameters simultaneously, rather than one at a time.

Given the set of variables and with the intervals of variation of each input, an SA was executed into the following analytic steps: (i) the testing model parameter configurations were sampled, (ii) the model output was evaluated for each parameterisation, (iii) finally model output is collected and used to compute sensitivity coefficients. FBA optimisations are mutually independent, hence (ii) was performed in parallel. The fbar R package provided a toolkit for FBA and related metabolic modelling techniques. We performed Sobol's variance-based SA relying on the *sensobol* R package (Puy *et al.*, 2022), which provides implementations to conduct variance-based uncertainty and sensitivity analysis. Given D parameters in the model, the function *sobol_matrices()* allows for $N * (2 * D + 2)$ independent random samples of the parameters space, where N is a user-defined parameter.
The method determines two kinds of sensitivity indices. The first-order indices S_i measuring the contribution to the output variance by a single model input alone:

$$S_i = \frac{\mathrm{Var}[\mathbb{E}(Y|Q_i)]}{\mathrm{Var}(Y)} \tag{S3}$$

where S_i is calculated according to the sensitivity of a single parameter Q_i on the output variance of the model. The numerator represents the variance of the expected value of the output Y given the parameter Q_i, while the denominator represents the variance of the output Y. The result represents the fractional contribution of the parameter Q_i to the output variance of the model. The total-order index S_{Ti} measures the contribution to the output variance caused by a model input, including both its first-order effects and all higher-order interactions:

$$S_{Ti} = \frac{\mathrm{Var}[\mathbb{E}(Y|Q_i)] + \sum_{j\neq i} \mathrm{Var}[\mathbb{E}(Y|Q_i, Q_j)]}{\mathrm{Var}(Y)} \tag{S4}$$

where the sensitivity of a single parameter Q_i on the output variance of the model, including the sensitivity due to interactions (covariance) between the parameter Q_i and all other parameters. Both the variance of the expected value of the output Y given the parameter Q_i and the variance of the expected value of the output Y given the parameters Q_i and Q_j; where Q_j represents all other parameters except Q_i.

SA implementation.

In our study, we implemented global SA to classify reactions in an FBA model based on their impact on model outcomes. This method, although computationally intensive, allows for an extensive exploration of input factors. We utilised Sobol's variance-based SA, combined with a random sampling scheme of parameters, to estimate the sensitivity indices of input parameters (Saltelli *et al.*, 2010).

Our approach involved generating a set of samples using Sobol's Quasi Random Numbers sequences, which were then used to compute the model's output for each sample (Saltelli *et al.*, 2010). We applied this method to the clostridioides reconstruction *Clostridium difficile* CD196, which required altering 186 boundary reactions and performing about 1.5 million FBA optimisations. We relied on the VMs+Storage services offered by the High-Performance Computing for Artificial Intelligence (HPC4AI) laboratory at the University of Turin. Due to the mutual independence of all linear programming optimisations, we offload computation to a parallel architecture with more than 5k cores. We leveraged the independence of FBA optimisations by distributing them across multiple cores where the parameter values were sampled using a uniform distribution, and the sample matrix was divided into chunks, each containing 12000 parameter configurations.

SA application.

Genome-scale metabolic networks encapsulate all enzymatic reactions encoded in a given genome. In our study, we focused on the metabolism of *C. difficile*, which comprises over 1300 biochemical reactions whose analysis remains challenging due to largely restrictive and undetermined information, such as kinetic parameters of rate laws and the concentration of chemical species.

In the *Clostridium difficile* CD196 metabolic network, there are a large number of reactions (186) that exchange with the model's external boundary. Estimating the value of each extracellular flux may not be feasible due to this large number. Therefore, it's crucial to quantify the influence of these constraints on the model outputs to determine the most sensitive inputs. In our specific scenario, this approach facilitated the exploration of the factors involved in the cross-talk between different components of the system, represented by varying levels of analytical detail.

We downloaded the metabolic reconstruction of *Clostridium difficile* CD196 from the GitHub repository hosting AGORA (Assembly of Gut Organisms through Reconstruction and Analysis). The repository contains the *Clostridium difficile* CD196 metabolic model for AGORA version 1.03 in MATLAB format. We manually edited the model by incorporating boundary reactions not included in the reference reconstruction. The reversible sink reaction for heme:

$$\mathrm{sink\_pheme\_c : pheme\_c <==> \emptyset} \tag{S5}$$

was added to enable the simulation of metabolic behaviour under changes in heme availability or genetic perturbations. By defining the heme inputs and outputs of the system, the heme sink reactions provide a framework for integrating heme dynamical detail into the model. Such an augmented model contains 1368 reactions (including 186 boundary reactions), 1005 metabolites, and 889 genes. We investigated the sensitivity of all the D=186 boundary reactions that allow the exchange of metabolites between the system being modelled. In this regard, we defined the parameter N equal to 2^13, resulting in $N \cdot (D + 2) = 1540096$ independent optimisations distributed across 24 cores. This global SA pipeline ranks boundary reactions based on sensitivity, aiding in the identification of metabolites and reactions sensitive to the objective function of an FBA model, specifically biomass maximisation.

GreatMod framework

GreatMod (`https://qbioturin.github.io/epimod/`) is a general modelling framework to simulate biological complex systems using an intuitive graphical interface for model construction and automatic derivation of the low-level mathematical processes characterising the system dynamics.

GreatMod is composed of three main modules. The first consists of a Java GUI based on Java Swing Class, called GreatSPN (Amparore *et al.*, 2016), which allows the user to draw models using a high-level graphical formalism called Petri Net (PN) and its extensions. The other two modules implement all the functionalities needed for the model analysis: (i) the R library *Epimod* (Castagno *et al.*, 2020) and (ii) Docker containerisation, a lightweight OS-level virtualisation.

Epimod provides a user-friendly interface for easy access to analysis techniques for (i) the derivation of the model's underlying mathematical processes (e.g., the system of ODEs), (ii) the model sensitivity analysis to reduce the search space associated with each unknown parameter, (iii) the model calibration analysis to adjust the parameters to obtain the expected behaviour with respect to the available data, and (iv) the model simulation to answer specific questions through what-if analysis and to derive new insights. Finally, the Docker containerisation of all implemented analysis techniques ensures results reproducibility and framework portability, as specified by the RBP guidelines (Kulkarni *et al.*, 2018).

The Harmonisation Paradigm

Harmonisation definition

The key to harmonising dynamic and metabolic models lies in identifying metabolites and reactions to be coordinated. This involves assuming (i) shared metabolites between the models, which represent resources produced or consumed by both, and (ii) boundary reactions that facilitate the sharing of these metabolites. Our goal is to minimise essential parameters requiring measurement and to pinpoint influential parameters crucial for harmonisation. By integrating global SA with ODE-based models, we address the non-linear relationships between the most sensitive variables, overcoming FBA's linearity limitations.

Fig. S1 displays the sensitivity coefficients for the top-ranked parameters, showing first-order (direct impact) and total-order (overall contribution) sensitivity coefficients. A consistency among total-order coefficients suggests potential linear relationships between variables. This global analysis identifies parameters that need further research to enhance our understanding and reduce uncertainties. The input variable with the highest total-order sensitivity is tryptophan flux (Trp_L), followed by teichoic acids ($DM\_teich\_45\_BS$), pantothenate ($pnto\_R$), and leucine (Leu_L). These findings highlight the importance of accurately defining flux boundaries for biologically relevant predictions.

SA results align with literature (Karasawa *et al.*, 1995), indicating that cysteine, isoleucine, leucine, proline, tryptophan, and valine are essential for *C. difficile* growth. Indeed, the most sensitive input variable is Trp_L, followed by Leu_L. Dipeptides Gly_Leu and Ala_Leu also rank highly in sensitivity, underscoring their importance for bacterial growth. Teichoic acids and pantothenate, while sensitive inputs are less relevant for investigating host-pathogen interactions in CDI within our framework.

By ensuring that key metabolites and reactions are accurately represented and coordinated between dynamic and metabolic models, the harmonisation enhances the biological relevance and accuracy of unified models. This is particularly important for understanding complex systems like bacterial growth and metabolism, which are influenced by a large number of interacting factors.

Also, the improved integration and SA lead to more robust biological model predictions. This has direct implications for studying metabolic pathways, growth conditions, and potential interventions in bacterial systems such as *C. difficile*. While the harmonisation primarily focuses on metabolic and dynamic model integration, it also highlights the lesser relevance of certain inputs for investigating host-pathogen interactions in CDI. This specificity ensures that the research remains focused on the most impactful areas, enhancing the practical applicability of the findings.

Harmonisation formalism.

We detailed information on essential amino acids (Cys_L, Ile_L, Leu_L, Pro_L, Trp_L, and Val_L) as ODEs, identified as the most influential input parameters. Using the GreatMod framework, we designed an Extended Stochastic Petri Net to facilitate information transfer between ODEs and the genome-scale metabolic network. This allowed the ODEs-based model to be fine-tuned to match the behaviour of the FBA model and experimental data.

The harmonisation formal definition requires first the definition of a generic dynamic model with n interacting elements and h possible events (some of them might represent reactions) in terms of the ODEs system:

$$dx_i(\nu) = \sum_{j=1}^{h} \phi_{i,j}(X(\nu), \nu)d\nu \qquad i \in \{1, \ldots, n\} \tag{S6}$$

where ν represents the time, $x_i(\nu)$ the number of the i-th element at time ν, $X(\nu)$ the vector of dimension n storing the number of each element at time ν, and $\phi_{i,j}(X(\nu), \nu) \in \mathcal{R}$ the occurrence velocity of the j-th event considering the i-th element (if negative then the i-th element is consumed by the event, otherwise it is produced).

Differently, a generic metabolic model with m metabolites and r reactions can be defined in terms of the FBA problem as in Eq.s S2.

Now the harmonisation between the two models is defined as follows. Defined F as the set of all the reactions in the FBA model, let $F^{bound} \subset F$ be the subset of the three types of boundary reactions: exchange, sink, and demand reactions.

Thus, a set of boundary reactions exists, $F^{shared} \subset F^{bound}$, such that each corresponding flux can be associated with an occurrence velocity in the ODEs system, representing the exchange of such metabolites. We report in the Supplementary Section S1.3 more details about how to define F^{shared}.

Mathematically, let M^{Ex} be the set of ODEs indexes representing the extracellular metabolites exchanged through the fluxes in F^{shared}. We can define an objective function $\mathcal{J}$ that maps an index from M^{Ex} into the index $\widehat{\mathbf{k}}$ of the respective flux index, $v_{\mathcal{J}(\widehat{\mathbf{k}})}$. Indeed, if there exists an exchanged metabolite without an ODE associated, i.e., $\exists \widehat{\mathbf{k}} \in M^{Ex}$ s.t. $\mathcal{J}(\widehat{\mathbf{k}}) = \emptyset$, then a new ODE has to be defined to represent the concentration of the metabolite exchanged through the flux $v_{\mathcal{J}(\widehat{\mathbf{k}})} \in F^{shared}$. Thus, the ODEs

associated with M^{Ex} will be characterised by a set of events that allows the connection between the starting ODEs model and the FBA model. So, the Eq. S6 becomes:

$$dx_i(\nu)/d\nu = \sum_j \phi_{i,j}(X(\nu),\nu) + \mathbf{1}_{(i\in M^{Ex})}\ v_{\mathcal{J}(i)}(\nu) \tag{S7}$$

where $\mathbf{1}_{(i\in M^{Ex})}$ is the indicator function that returns 1 only if the index $i \in M^{Ex}$. Furthermore, the fluxes are time-dependent since are conditioned to the FBA model which is continuously solved with respect to the state of the ODEs system at time ν:

$$\begin{aligned}
&maximize && f_{obj}(v), && \text{(S8)}\\
&\text{subject to} && && \\
& && S\cdot v = 0, && \\
&v_k \notin F^{shared} && v_k^{min} \le v_k \le v_k^{max}, && \\
&v_k \in F^{shared} && f_{lower}(v_k,\hat{x}) \le v_k \le f_{upper}(v_k,\hat{x}), && \\
& && v_k \in \mathbb{R}, \qquad \forall k = 1,\ldots,\ r. &&
\end{aligned}$$

The functions f_{lower} and f_{upper} represent the lower and upper bounds in which each v_k flux varies, which can be defined as a linear transformation of the respective exchanged metabolites concentration (i.e., $x_{\mathcal{J}^{-1}(k)}$) when $i \in M^{Ex}$.In such a manner, the concentrations from the ODEs could be used as flux constraints because they represent the availability of the metabolites in the environment and therefore the uptake limit. Usually, the upper constraint is set only if a growth limit has to be defined, otherwise it should be infinity (see the Supplementary Section S1.4 for more details).

Harmonisation definition exploiting ESPNs as meta-formalism.
The ESPN formalism becomes a meta-formalism that allows us to generalise and graphically harmonise the dynamic model with metabolic models by exploiting *general transitions*.

Thus, given an FBA model as Eq. S2, we refine the set of general transitions, called T_g, into two disjoint subsets T_g^{FBA} and $T_g^{\neg FBA}$, so that i) $T_g^{FBA} \cup T_g^{\neg FBA} = T_g$, and ii) $t \in T_g^{FBA}$ iff its intensity is obtained by solving the FBA model.

Let us observe that, by definition, the transitions' intensity has to be a positive number, therefore if reversible reactions are considered in F^{shared}, then two different transitions, denoted with *_in* if the estimated flux is negative and *_out* if it is positive, have to be defined (an example is shown in Fig. 1). Thus, we can infer that

$$\begin{aligned}
\forall v_k \in F^{shared} &\implies \exists\ t_{v_k,in}\ \wedge\ t_{v_k,out} \in T_g^{FBA} \\
\text{s.t.} \qquad & f_{t_{v_k,out}}(\nu,\hat{x}) = \mathbf{1}_{(v_k\ge 0)} v_k\ \wedge \\
& f_{t_{v_k,in}}(\nu,\hat{x}) = -\mathbf{1}_{(v_k\le 0)} v_k
\end{aligned} \tag{S9}$$

where, $f_t()$ is the intensity associated with the general transition t, $t_{v_k,in/out}$ the general transition representing the flux v_k, and $\mathbf{1}_{(\cdot)}$ is the indicator function that returns 1 only if the input condition is satisfied.
Hence, the instantaneous changes of tokens $x_i(\nu)$ in the ith place at time ν expressed in the Eq. S7 can be rewritten as follows:

$$\begin{aligned}
\frac{dx_i}{d\nu} = &\sum_{t\in T_{ma}} \phi(t,\hat{x})L(p_i,t) + \\
&\sum_{\overline{t}\in T_g^{\neg FBA}} f_{\overline{t}}(\nu,\hat{x})L(p_i,\overline{t}) + \\
&\sum_{\overline{t'}\in T_G^{FBA}} f_{\overline{t'}}(\nu,\hat{x})L(p_i,\overline{t'}).
\end{aligned} \tag{S10}$$

Thus, the $f_{\overline{t'}}(\nu,\hat{x})$ associated with a general transition $\overline{t'} \in T_g^{FBA}$ must be defined as in Eq. S9, with the fluxes v conditioned to the FBA model (Eq. S8) adding the dependence of the input places to the functions defining the bounds.

Reactions identification

Let us consider the FBA model defined in Eq. S2 in the main paper. Thus, we suggest following a few simple rules to identify the reactions which will characterize the set F^{shared} as being efficiently coupled with the system of ODEs, as shown in Eq. S7 (main paper).

Considering boundary reactions.
Firstly, we need to filter the set of all the fluxes of the FBA model, namely F, to consider the reactions that can be actually used to define F^{shared}, i.e., the *boundary reactions*. Among all the reactions, we strongly suggest using only the boundary ones for two reasons: (i) to maintain consistency with the fundamentals of our paradigm, and (ii) not to violate the balance assumption of the

FBA model. The paradigm has to connect two modelling approaches with their assumptions and rules, simulating two different system layers which share a given number of resources (e.g., metabolites). In particular, the FBA model is constrained by the steady state of the system, which supposes that there are no changes in any metabolite concentration in a specific time window. Therefore, by defining an ODE modelling the metabolite concentration over time of the FBA model, it is straightforward to see that the steady-state assumption of the FBA model does not hold anymore. Differently, the boundary reactions represent by definition the connection of the metabolic model with an extracellular environment, delineating in such a manner the perfect bridge between an ODE representing the shared metabolite outside the metabolic model, which acts as its resource by modifying dynamically the reaction constraints.

Sensibility of the model.
Among the boundary reactions, the ones with a greater sensibility to perturbations in their constraints play a central role in the connection between the two modelling approaches. Given that the ODEs system is connected to the FBA model in terms of the fluxes constraints, which are defined by the functions $f_{lower}(\cdot)$ and $f_{upper}(\cdot)$, it is straightforward to consider reactions where small perturbations in the concentration of the shared metabolite affect the FBA solution. Otherwise, the two approaches could be solved independently without dynamic communication that might improve and change the analysis. Therefore, we suggest solving multiple times the FBA model varying the constraints of the boundary reactions to identify the ones that potentially will be the ones constituting F^{shared}.

Reaction gap filling.
Indeed, the knowledge of the system under study and the aim of the model plays a key role in the selection of these reactions, which can be exploited either to further filter, if just a few reactions are of interest, or to expand F^{shared} with new reactions that are not present in the metabolic model. Considering the latter, the model could be either manually refined through the so-called gap-filling approaches. Reaction gap filling is a computational technique introduced in (Karp *et al.*, 2018), by which it is possible to add a set of reactions to genome-scale metabolic models to obtain high-accuracy models. Gap filling completes what are otherwise incomplete models that are derived from annotated genomes in which not all enzymes have been identified. Thus, by adding specific boundary reactions to the FBA model, which will be part of F^{shared}, it is possible to manually define exchanged metabolites that will connect the ODEs and FBA models.

Constraints definition.

The functions f_{lower} and f_{upper} represent the lower and upper bounds in which each v_i flux varies, which may depend on the marking of the transition input places. Indeed, as reported in (Covert *et al.*, 2008), several types of metabolic flux constraints can be used to define the f_{lower}/f_{upper} functions:

1. **irreversibility constraints**, where the lower bound of the reaction is set to zero for reactions that can only proceed in the forward direction (Covert and Palsson, 2002);
2. **environmental constraints**, where the maximum flux through an exchange reaction is limited by the amount of substrate in the culture medium (Varma and Palsson, 1994);
3. **transport constraints**, which are represented as a maximum substrate uptake[2];
4. **regulatory constraints**, where the flux through an enzyme is restricted by the expression of the corresponding protein(s) (W. Covert *et al.*, 2001);
5. **ODE matching constraints**, where the fluxes are specified by the ODE model.

The first three types of constraints are derived from the literature (and it is the classical manner to define the constraints), so the functions can be expressed as $f_{lower/upper}(v_i, \hat{x}) = k_i^{lower/upper}$, where $k_i^{lower/upper} \geq 0$ are constants. Indeed, when the flux does not depend on the ODEs values, then the $\hat{x}$ dependency can be omitted.
The regulatory constraints can be obtained by encoding in the function associated with the general transition a Boolean regulatory model, i.e., a set of Boolean logic equations that involve restricting expression of a transcription unit (sequence of nucleotides in DNA that codes for a single RNA molecule) to the value 1 if the transcription unit is transcribed and 0 if it is not. Similarly, the presence of an enzyme or regulatory protein, or certain conditions inside or outside of the cell, may be expressed as 1 if the enzyme, protein, or a certain condition is present and 0 if it is not. We decided not to consider this type of constraint but to focus on the ones that depend on the ODEs characterising the part of the ESPN which is not considered in the FBA. In this case, as shown in (Covert *et al.*, 2008), the upper and lower bounds of the FBA fluxes depend on the entities modelled from the ODE system, and they are settled equally to the corresponding rate calculated by the ODE model. In such a manner, the concentrations from the ODEs could be used as flux constraints because they represent the availability of the metabolites in the environment and therefore represent the uptake limit. Usually, the upper constraint is set only if a growth limit has to be defined, otherwise, it should be infinity.

Synchronisation.

Since we are implementing a hybrid model technique that combines two different algorithms for solving differential and algebraic systems, we must define rules to synchronize these techniques. In particular, we define the ODEs system as the model leading the simulation, while the LPP characterizing the FBA is exploited to calculate the value of some components of the ODEs system.

[2] Typically the rate of uptake of nutrients is dictated by availability (a nutrient that is not present cannot be absorbed), concentration and diffusion constants (higher concentrations of quickly-diffusing metabolites are absorbed more quickly).

Thus, according to this, we have to decide how many times and under which hypothesis the LPP has to be solved, and in this manner, the respective components in the ODEs system are updated. In (Covert *et al.*, 2008), the authors proposed a new type of DFBA, called integrated FBA, in which they integrated the FBA metabolic network with a Boolean transcriptional regulatory network and an ODE model. In particular, their simulations are characterized by consecutive numerical integrations of the ODE model and solution of the LPP, whose interaction depends on some common metabolites and variables. In this context, they suggest choosing the length of each time step to be large enough that the FBA assumption (the concentrations of internal metabolites are time-invariant) holds, and yet small enough for the ODE model to calculate the system dynamics without accumulating numerical error.
Starting from this consideration, we defined a more general way to call the LPP during the ODEs solution without stopping it. In detail, we decided to solve the LPP only if the differences between all the input places of the general transitions modelling the FBA at two consecutive times ν_i and time t_{i+1}, with $0 \leq \nu_i < \nu_{i+1} < \nu_{final}$ (ν_{final} is the final time selected for the ODE solution) is greater than $\epsilon > 0$. Mathematically, this can be expressed as follows (starting from the Eq.s S10 in the main paper):

$$\begin{aligned} dx_i(\nu) = & \sum_{j=1}^{n_{T_{ma}}} \phi(t^*_j, \hat{x}) S(p_i, t^*_j) d\nu + \sum_{j=1}^{n_{T_g} - n_{T_g^{FBA}}} f_{\bar{t}_j}(\nu, \hat{x}) S(p_i, \bar{t}_j) \\ & + \sum_{j=1}^{n_{T_G^{FBA}}} \left[(1 - \mathbf{1}_{\{cond\}}) f_{\bar{t}_j}(\nu, \hat{x}^{last}) + \mathbf{1}_{\{cond\}} f_{\bar{t}_j}(\nu, \hat{x})\right] S(p_i, \bar{t}_j) d\nu \\ & \forall i \in \{1, \dots, n_P\}, \end{aligned} \tag{S11}$$

where $\mathbf{1}_{\{cond\}}$ is the indicator function which is 1 if the condition expressed in *cond* is true, otherwise is 0. Such condition can be defined as follows:

$$cond = \begin{cases} TRUE & \text{if} \quad \bigvee_{x_p:\ x_p \in \hat{x}_{FBA}} |\Delta x_p| > \epsilon, \\ FALSE & \text{otherwise}, \end{cases} \tag{S12}$$

where $\hat{x}_{FBA} = \{x_p : p \in^\bullet \mathbf{t} \ \wedge \ t \in T_g^{FBA}\}$ is the set of the markings of all the input places of the general transitions modelling the FBA, and Δx_p is the discretisation of the absolute difference of the ODEs solution, i.e. $\Delta x_p = x_p^{last}(\nu') - x_p(\nu)$. Where, $x_p^{last}(\nu')$ represents the marking of $x_p \in \hat{x}_{FBA}$ at the last time ν' at which the FBA was calculated, with $\nu' < \nu$. While $x_p^{last}(\nu')$ is the 'present' marking of$x_p \in \hat{x}_{FBA}$ at the time ν. Therefore, the LPP is solved only if there exists a difference (defined by ϵ) between the only variables that could change the results of the FBA since the constraints of the flux associated with a specific general transition $t \in T_g^{FBA}$ might depend on its input places marking. Otherwise, there would not be any important differences in the fluxes obtained from solving the FBA characterized by the same constraints (the only parameters of the FBA model that vary and depend on the ODE model).
Let us observe that in cases where an empty feasible region is detected for the FBA resolution, the solver we implemented in the R package "*epimod*" flags the issue by providing some diagnostic information.

Escherichia coli unified model

An optimal test bed for our *UnifiedGreatMod*is given by the simulation of the carbon catabolite repression phenomenon exhibited by *E. coli* (Ammar *et al.*, 2018). The bacterium adapts its metabolism to the environment's available carbon sources (i.e., D-glucose or lactose). The LacZ system plays a crucial role in *E. coli*'s adaptive response to changes in available carbon sources. The role is to ensure that the cell expends energy on producing enzymes encoded by the lac operon only when necessary. To model this system at a genome-scale, we establish gene-protein-reaction (GPR) associations, connecting genes, proteins, and reactions via the flow of our novel modelling paradigm.

Overview of the modelling approach.

In the iML1515 model, the lacZ gene encodes the enzyme beta-galactosidase, which catalyses the conversion of Lactose into D-glucose. The gene and the corresponding beta-galactosidase associated reaction are explicitly included (see Fig. S2D) in the metabolic network. By leveraging GPR associations, we employ Flux Balance Analysis (FBA) to predict metabolic fluxes. This allows us to simulate and study the activity of the LacZ system within the broader context of the metabolic network. Finally, integrating the regulation of the lacZ gene expression into our model provides a more accurate representation of the bacterial metabolic response to varying environmental conditions. For example, the model accounts for the fact that the lacZ gene is expressed only when lactose is present and D-glucose is absent.

Our approach combines experimental data, computational techniques, and environmental context to create a comprehensive computational translation considering a holistic view of biological systems. In this example, the unified model harmonises the diverse interacting domains of knowledge. We apply constraints derived from experiments to account for specific conditions. These constraints represent both intra- and extracellular factors, including (i) environmental restrictions, (ii) enzyme capabilities, and (iii) biomass maintenance needs. To implement these constraints, we set reaction flux bounds—defining lower and upper limits for each reaction. This ensures that our model adheres to the physiological boundaries observed in the biological system.

We develop a computational pipeline accounting for differential reaction activities and leveraging transcriptomics data. By mapping expression patterns of metabolic enzymes directly to changes in metabolic fluxes, we gain insights into the dynamic behaviour of the system. Specifically, we utilise data from *E. coli* obtained via the commercial microarray platform Affymetrix GeneChip system (Liu *et al.*, 2005) as the expression data source. The expression data originates from MG1655 cells cultivated aerobically in MOPS minimal medium, supplemented with 0.1% glucose (Bergholz *et al.*, 2007) representing the cell cultivation conditions. By tuning the bounds associated with exchange reactions, we simulate the availability of various nutrients in the environment. This integration allows us to capture both the internal metabolic state of the cell and the external conditions under which it grows.

Methodology

We used our computational workflow to construct the model of the metabolic system. The first step involved defining a mechanistic model and using Petri Net formalism to model the time dynamics of target metabolites. We then identified the metabolic network using the COBRA model of the *E. coli* K-12 strain MG1655. The next step was to identify the components of the metabolic model that would be coupled, which could include enzymes and metabolites involved in target metabolic reactions. After coupling the identified metabolic network, components, and mechanistic model, we converted metabolic reactions into an irreversible format to facilitate exchange reactions in Petri Net formalism. This involved splitting reversible reactions into two components and extending reaction identifiers with *in* or *out* to preserve original direction information.

The unified model, consisting of two places and six transitions, accounted for the metabolite concentrations required by the corresponding ODE model. The biochemical reactions of interest in the batch media were described by the Petri Net model, where transitions represented the inflow of fresh nutrients from the external environment. Finally, we used the coupled model to run simulations and predict system behaviour under various conditions. The model was used to demonstrate the direct effects of carbon supplementation on glucose and lactose consumption rates and bacterial biomass production in a biologically active environment. These rates were defined to model different carbon source administration regimes in a real-world batch culture scenario.

Results

By following this approach, we gain insights into the dynamic behaviour of the LacZ system and its impact on overall metabolic processes. This study provides insights into the dynamics of the LacZ system and its influence on the metabolism of *E. coli*. We simulated different carbon source supplementation scenarios for a fed-batch. These include *constant feeding* (steady glucose and lactose rate), *linear feeding* (increasing glucose and lactose rate over time), *pulsed feeding 60* and *pulsed feeding 150* (adding glucose and lactose in short cycles), and *blank* (no glucose and lactose added). The prediction of sugar levels (Fig. S2A) shows a variation in exchange reaction activities (Fig. S2B) following the nutrient administration.

FBA resolution also estimates biomass flux (i.e. the cellular growth rate) under the given conditions (Fig. S2C). A biomass flux of about 0.65 mmol/gDW*h means that each gram of dry weight of the *E. coli* strain K-12 MG1655 is producing 0.65 mmol of biomass per hour. This value measures the metabolic activity estimated as the ability to convert the nutrients in the medium into new cell material. In a culture in the middle of its logarithmic growth phase, the estimated growth rate resembles the exponential growth phase (coli Genome Project University of Wisconsin–Madison, 2023). After the exponential growth phase, the cells have already utilised a substantial portion of the available nutrients, leading to a deceleration in their growth rate. *E. coli* efficiently uses glucose before its depletion due to its preferential metabolic pathway. The genome-scale metabolic network iML1515 includes a subnetwork for the uptake and metabolism of D-glucose and lactose (Fig. S2D), illustrating the dynamics of the lac operon. This subnetwork shows the transportation of D-glucose and lactose from the extracellular space to the periplasm and cytoplasm, where lactose can be cleaved into D-glucose by beta-galactosidase. The LacZ system, represented within this subnetwork, enables the cell to switch between D-glucose and lactose metabolism. This switch is governed by the availability of the sugars, with gene expression data and nutrient concentrations dynamically adjusting the bounds of key reactions (Fig. S2E).. Once glucose levels drop, the LacZ system is induced, enabling *E. coli* to use lactose as an alternative carbon source. The LacZ activity is constrained based on glucose availability to simulate the metabolic shift. When glucose is present, the LacZ system is repressed, but upon glucose depletion, the LacZ system is activated, allowing *E. coli* to use lactose after glucose depletion. This abrupt activation reflects the observed diauxic shift in *E. coli* metabolism. The switch on of lactose metabolism takes place around time 10 hours characterised by an increase of the bounds of reactions involving lactose uptake and beta-galactosidase activity when glucose levels are low and lactose is available and a fluxes rerouting through the subnetwork. This involves enhancing the transport of lactose from the extracellular space to the periplasm, the conversion of lactose to D-glucose in the periplasm, and the subsequent transport of D-glucose to the cytoplasm. (Fig. S2E, plot 2 and plot 4).

modelling host and drug responses to Clostridium difficile infection.

Metabolic Basis of C. difficile pathogenesis

Intestinal epithelial cells ensure the transport of dietary amino acids through a process of uptake across the apical membrane, diffusion through the cytoplasm, and release via the basolateral membrane to the portal vein (Argiles and Lopez-Soriano, 1990). *C. difficile* is a minor member of the gut microbiota that often lives harmlessly in the small intestine so long as the ability of the gut microbiota to resist bacteria colonisation is maintained. However, some circumstances allow *C. difficile* overgrowth and disrupt the barrier function of the intestinal epithelium via enterotoxins production. The regulatory anti-starvation mechanisms

are also involved in toxin production, corroborating that *C. difficile*'s pathogenesis is a blend between bacterial metabolism and the nutritional status of the environment.

The physiological effects of toxins involve mainly necrotic cell death increasing vascular permeability, affecting amino acid balance, and causing haemorrhaging. This might create a nutritional gut niche derived from the unleashing of nutrients from the host. There are some nutrients critical for efficient growth, which lack can trigger drivers of metabolic stress (Neumann-Schaal *et al.*, 2015). Toxin-mediated inflammation alters the lumen metabolic pool of key amino acids (e.g. proline, leucine, isoleucine, valine, tryptophan and cysteine) establishing a direct link with gut environmental nutrient availability and to supporting increased bacterial growth (Edwards *et al.*, 2014).

Physiological alterations during antibiotic perturbation

Guidelines about the awareness, diagnosis and treatment of CDI are available. However, several studies have reported treatment failure after an antibiotic course with varying extents of drug resistance (Debast *et al.*, 2014). Given antimicrobial agents' multifactorial action in different conditions, therapies informed by metabolic mechanisms of action will enable options with improved safety and efficacy. The metabolic alteration related to drug activation and deactivation is particularly suited to system-wide analysis that integrates multiple solution techniques. Therefore, we suggest a new computational approach to systematically investigate deregulated biochemical pathways on the landscape of metabolic changes concurrently with drug exposure.

The mechanism of action of different antimicrobial agents used to treat CDI is partly known. For example, nitroimidazoles require the reduction of the nitro group, which results in short-lived reduction products that oxidize DNA, followed by quick death in susceptible cells (Dingsdag and Hunter, 2017). The spectrum of activity depends on the selective action of the drugs on the specs of anaerobic metabolism. Nitroimidazole, changing into a component of the electron transfer system between pyruvate and hydrogenase, works as an electron siphon interrupting the electronic flow. The reduction product of the drug is directly responsible for getting cells killed. A sensitive organism undergoes several disrupted physiological mechanisms or effects together in a synergistic fashion.

Drug detoxification response modulated by micronutrients

We discuss novel findings regarding how micronutrients modulate the responses of both the host and *C. difficile* during infection. Various physiological conditions can alter micronutrient availability, contributing to infection susceptibility. Micronutrients necessary for regulating bacterial homeostasis may be derived from the inflammatory environment. Notably, micronutrients such as iron-coordinated heme, besides being indispensable for bacterial metabolism, function as redox-active molecules and redox stress defence factors (Knippel *et al.*, 2018).

The inflamed gut contains numerous environmental oxidative stressors that can act as selective pressures, driving the evolution of mechanisms for oxidative stress protection. It is plausible that *C. difficile* acquires heme exogenously during infection, likely from the host due to toxin-mediated damage to the gastrointestinal epithelial layer, and endogenously from the diet.
As an obligate anaerobe, *C. difficile* faces significant oxidative stress, particularly due to targeted antimicrobial therapy. The physiological effects of antibiotics used to treat anaerobic bacterial infections (e.g., nitroimidazoles) depend on the bacterial detoxification systems. This suggests that the acquisition of heme and the ability to withstand oxidative stress are crucial factors in the evolution of antibiotic resistance in *C. difficile*. These adaptive mechanisms enable the bacterium to survive in the hostile environment of the inflamed gut and resist antimicrobial therapy. Novel adaptive mechanisms may rely on disrupted metabolic pathways and reprogrammed bacterial metabolism, with necessary micronutrients for bacterial homeostasis derived from the inflammatory environment.

UnifiedGreatMod application: C. difficile infection

C. difficile strain CD196 GSMM was originally coded in the AGORA/MATLAB (1.03 version) format (Magnusdottir *et al.*, 2016), comprising over 1300 biochemical reactions, and then elaborated using the R functions available at `https://github.com/qBioTurin/epimod_FBAfunctions.git`, designed to integrate FBA-based models within the R library. The computational cost of these processes is dependent on the size of the metabolic network. For the *C. difficile* strain CD196, it was observed that the execution time for the FBA model run was approximately 100 seconds. In terms of memory usage, the process consumed approximately 0.6 MB
Parameter configurations obtained from sensitivity analysis of the unified model, showing three distinct sets identified based on their proximity to median trajectories of $\text{IECs}_{t=20}$ counts (Table S3). The parameters represent: Detox (antibiotic detoxification ability), Death4Treat (antibiotic susceptibility), and IECsDeath (bacterial toxicity) corresponding to the behavioural patterns observed in the main analysis (Figure 2 B left).
The metabolic model is parametrised using data of gut microbial metabolism and nutrition (Noronha *et al.*, 2018). These nutritional data capture interactions in the context of human diet and microbiology. The coupling of ODEs and metabolic model dropped within one of the following scenarios: (i) when rates of ODEs and GSMMs reactions exactly match, the flux in the metabolic model was fixed to that in the dynamical model and represented as events using the ESPN description (ii) the subset of shared metabolites between both models are used for inbound communication in the metabolic model as constraints set.
We presented a detailed example to illustrate the formalised representation method. The cellular component and molecular interactions involved in the CDI are defined by the model shown in (Fig. S3). From top to bottom, the model is organised

into six modules: (i) *Blood vessel*), (ii) (*IECs*, (iii) *Lumen Env*, (iv) *C. difficile cells dynamics*, (v) *C. difficile metabolic network* and (vi) *Drug* modules.

We describe these modules in detail hereafter and report the mathematical information regarding the rates associated with each module's transitions. By the definition of ESPN formalism, transitions that do not follow the Mass Action (MA) law are modelled as general transitions. In detail, the speed of general transition $t \in T_g$ is defined by a function $f_t(\hat{x}(\nu), \nu)$, where $\hat{x}(\nu)$ represents the vector of the average number of tokens for all the transition input places at time ν and t is the transition. Differently, if $t \in T_{ma}$, the velocity is defined as the MA law: a constant rate associated with the transition t, namely $\lambda(t_i)$, multiplied by the marking of all the input places of t powered by the cardinality of the arc, i.e., $\phi(t, \hat{x}) = \lambda(t) \prod_k \hat{x}_k^{I(p_k,t)}$. Let us observe that $x_{place}(\nu)$ denotes the average number of tokens in the place called *place* at time ν.

Blood vessel module.

The transport of dietary amino acids into the bloodstream takes place across intestinal epithelial cells. This module can dynamically describe the transepithelial amino acids transported into the circulation following protein digestion and absorption. Most of the amino acids absorbed by epithelial cells (over 95%) are released in the bloodstream, whereas only a minor fraction is for cellular self-maintenance.

Transport transitions (referred to as T_{amino_e} remove ingested amino acids taken up from the gastrointestinal lumen and add the amount in the bloodstream. We gave these transition rates proportional to the concentration of metabolites that served as inputs to the transitions multiplied by a factor α (Table S1), assuming the nutrition amount restrained by epithelial cells.

IECs module.

The gut epithelium is the tissue comprising the intestinal epithelial cells. In this perspective, *IECs module* models intestinal epithelial cells dynamics and the absorption of nutrients by these cells. This module represents the processes related to altering the gut metabolic pool, such as the roles in nutrient absorption and death-induced nutrient release by IECs.

The state change refers to the transition $T_{amino} \in \{T\_pro\_L\_e,\ T\_leu\_L\_e,\ T\_ile\_L\_e,\ T\_val\_L\_e,\ T\_trp\_L\_e,\ T\_cys\_L\_e\}$, which removes at a constant rate from the system specific quantity of amino acids depending proportionally on correspondent input place $amino \in \{pro\_L\_e,\ leu\_L\_e,\ ile\_L\_e,\ val\_L\_e,\ trp\_L\_e,\ cys\_L\_e\}$. The speed of transitions is also proportionally related to the number of intestinal cells. These transitions are prevented from consuming the token from the *IECs* place, where each token represents a viable and functional IEC. The transition-associated functions are defined as:

$$\lambda(T_{amino}) = \frac{\alpha}{x_{IECs}(0)} \tag{S13}$$

where α is the proportion of ingested amino acid taken up from the gastrointestinal lumen by enterocytes and transferred to the bloodstream. The term $x_{IECs}(0)$ is the initial number of cells forming the intestinal epithelium.

The initial number of cells was derived as follows. We consider that a single mL of cell culture (C_{IECs} equal to $2 * 10^5\ cell/mL$) was plated in each Snapwell insert (well radius equal to 1.13 cm^2) and cultured for two weeks, as described by (Anonye *et al.*, 2019). The European Collection of Authenticated Cell Cultures (ECACC) reported that Caco-2 cells reach confluence after four days when seeded at $2 * 10^5\ cell/cm^2$ and since their doubling time is 84 h, we calculate that at the confluence, within a Snapwell insert, the number of IECs is $4.57 * 10^5$ cells.

Dietary heme is a crucial source of iron which is taken up into IECs and degraded by heme oxygenases. Then heme iron translocates across the cytosol and is finally released into circulation as the same pathway for non-heme iron. Heme iron absorption is far more efficient than non-heme iron absorption. T_{pheme_e} transition models the heme absorption. This function is defined as:

$$\lambda(T_{pheme\_e}) = \frac{\beta}{x_{IECs}(0)} \tag{S14}$$

where β is the overall heme absorption yield. It is reasonable to expect that the overall dietary heme iron absorption yield is approximately 15–30% (Monsen, 1988).

The impact toxin harms on the mucosal intestinal response is displayed through its effects on epithelial cell death. *C. difficile* interaction with the single layer of intestinal epithelial cells leading to mucosal damage is modelled through the transition *IECsDeath*. This event represents IECs death. This transition is defined to represent the non-invasive pathogen features of *C. difficile* and the resultant toxin-mediated damage. Toxin production, which predominantly occurs during the stationary phase, is dependent on the bacterial amount. Therefore, *IECsDeath* velocity is proportional to the two input places *IECs* and *CD*:

$$\lambda(IECsDeath) = \delta \tag{S15}$$

where δ is the number of host cells a single bacteria can kill. The initial marking for the place *Damage* equals to zero, indicating that simulations start with full membrane integrity.

Upon IECs death, a specific amount of amino acid is released in the lumen per dying cell. When IECs defoliate, amino acids deposited as protein are lost into the intestinal lumen. Therefore, we can evaluate the amount of amino acids released upon biomass wasting. For dividing cell types, the generic human biomass reaction available in human metabolic reconstruction is formulated taking biomass precursor as input and the biomass supermetabolite as output (Thiele *et al.*, 2013). Biomass constraints are added

to a biomass reaction by defining stoichiometric coefficients for each biomass precursor. Stoichiometry represents the intracellular amino acid quantity released in the extracellular environment upon each cell's death. The arcs multiplicity was then computed given the amino acidic proportion constituting the prototypical human cellular biomass. Arcs connecting transition *IECsDeath* with output place *amino* is associated with multiplicity values detailing the number of tokens provided to target places:

$$\eta_{amino} = DW_{IEC}\frac{S_{amino}}{M_{amino}} \tag{S16}$$

where η_{amino} represents the quantity (arc's multiplicity) of amino acid injected in the output places *amino*. Each biomass unit lost several tokens, corresponding to the arc's multiplicity. If an amino acid residual has molar mass (M_{amino}), we can now convert the proportion into molar units.

Lumen Env module.
The gut lumen micro-environment is where the relations between bacterial colonizìsation, nutrient uptake and consumption occur. In the intestinal tract, this module models the toxin-mediated inflammation and the dietary intake of nutrients. This module allows for quantifying the gut metabolic pool value (i.e. essential amino acids and the micronutrient heme), which is the source of subsistence of bacterial growth during infection.
Minimal requirements of amino acids were previously determined for different strains (Karasawa *et al.*, 1995). Cysteine, isoleucine, leucine, proline, tryptophan and valine were essential amino acids for the growth of *C. difficile*. Each amino acid is compartmentalised into environmental units, such as the extracellular space. The overlapping reactions between the ODE-based model and the metabolic model across this module are the exchange reaction to represent the intake (of efflux) of essential amino acid, *amino*, in the extracellular environment (see *C. difficile metabolic network* module).

The *Lumen Env* comprises 7 places and 11 transitions. The initial amount of amino acids was measured by (Adibi and Mercer, 1973) in the lumen of healthy human volunteers, except for tryptophan and cysteine whose concentrations were unknown. These values were inferred considering amino acid residual frequencies described in (Dyer, 2010), as explained below.
Aminoacidic composition is a type of basic feature of a protein sequence, which includes 20 discrete numbers, each representing the occurrence frequency of each of the 20 native amino acids in a protein sequence, respectively. Regarding the sum of all 20 encoded amino acids, the initial amount is given by the following relation:

$$\sum_{A=1}^{20} X_A f_A = 1 \tag{S17}$$

where f_A is the expected frequencies of amino acids, and X_A expected concentrations.
Transitions $D\_pro\_L\_e$, $D\_leu\_L\_e$, $D\_ile\_L\_e$, $D\_val\_L\_e$, $D\_trp\_L\_e$, $D\_cys\_L\_e$ add to the system specific quantity of amino acid at a constant rate. Our model included the diet in the ODE-based model of the European Average diet. The nutritional data are collected at `https://www.vmh.life` (Noronha *et al.*, 2018).
Consequently, nutritional flux units are then defined as:

$$\lambda(D_{amino}) = \frac{\Delta}{24} d_{amino} \tag{S18}$$

where $\lambda(D_{amino})$ is the gross diet flux for amino acid residual *amino*, and d_{amino} is the nutritional flow for amino acid per person per day. We had to adapt available personal nutritional information to resize the nutritional flow to the model's desired scale. For 1 mL of volume considered, we introduced Δ as metabolite flow scaling factor. The parameter's value was set considering dietary flow d_{amino} at steady-state. We scaled to the amounts ($mmol$) as follows:

$$\Delta = \frac{1}{(1-\alpha)}\frac{Mean(X_A)}{Mean(d_{amino\_e})} \approx 10^{-4} \tag{S19}$$

where α refers to the proportion of amino acid removed by the gut absorption activity. The factor Δ essentially converts the dietary intake rate from a per person scale (mmol/hperson) to a per mL scale (mmol/hmL), assuming that the concentration of amino acids in the gut is equal to the ratio of the concentration to the intake rate. This is a reasonable assumption under the assumption that the body is in a state where the intake and removal of amino acids are balanced, resulting in a stable concentration.
The transition D_pheme_e models heme influx from nutrition is defined as:

$$\lambda(D_{pheme\_e}) = \frac{\Delta_{heme}}{24} d_{pheme\_e} \tag{S20}$$

We chose to use heme as a significant model component as the inflammatory tissue damage causes the liberation of high concentrations of host heme at infection sites. The initial amount of heme, $x_{pheme\_e}(0)$, was set as reported by (Hopp *et al.*, 2020). Analogously, to scale the dietary intake rate from mmol/h per person to mmol/h per mL (the volume unit considered in our model), we introduced a conversion factor for heme, denoted as Δ_{heme}.

Blood loss depends upon the integrity level of the IECs layer. Subsequently, red blood cells lyse once in the intestinal lumen, resulting in an abundance of extracellular heme, which is released from haemoglobin derived from ruptured erythrocytes under hemolytic conditions. Toxins cause severe damage to the colon's intestinal epithelial layer, resulting in inflammation and bleeding. Free heme released from erythrocyte after intestinal haemorrhage causes an increase in intracellular free heme, this event is modelled

by the transition *Inflam*.

IECs layer integrity could be monitored by measuring the transepithelial electrical resistance (TEER). The increase in host cell-associated bacteria was experimentally observed with a decrease in TEER by (Anonye *et al.*, 2019), suggesting a disruption event of the intestinal epithelial barrier. The event *Inflam* represents several processes related to toxin-mediated epithelial integrity disruption. Since the elevation of inflammatory cytokines and chemokines, capillary diameter and blood flow increase allowing the extravasation of red blood cells. Consequently, the established inflammatory environment causes cytoskeleton rearrangement and membrane instability in red blood cells, which become more rigid and undergo lysis-releasing heme (Gutierrez *et al.*, 2021). The heme-releasing reaction velocity modelled through the $Inflam$ general transition is defined by the following function:

$$f_{Inflam}(\hat{x}(\nu), \nu) = G\frac{x_{Damage}(\nu)}{x_{IECs}(0)}\left(\frac{\pi R(\frac{D_v}{2})^2 \times \rho \times Q \times H_{rbc}}{4I}\right), \quad \text{(S21)}$$

where G represents the grade of fenestration due to inflammation, R is the unbranched venules radius, Q is the red blood cell velocity, ρ erythrocytes density in human blood, D_v is the diameter of a villus blood vessel, and H_{rbc} is the amount of heme molecules within a erythrocyte. The villi of the small intestine project into the intestinal cavity, greatly increasing the surface area for food absorption and adding digestive secretions. Each villus is supplied with blood by 2 vessels, 1 venule, and 1 arteriole. I is the villus surface area, calculated as $I = \pi \times W \times L \times m$, where W is the villus width and L is the villus height, and m is the surface amplification due to microvilli (Solis *et al.*, 2005).

Bacteria have developed several strategies to capture heme and utilise the iron within the porphyrin ring. Utilisation of exogenous heme by bacteria involves the binding of heme to the cell surface receptors, followed by the transport of heme into cells. This reaction is mediated by the heme transport system represented by the transitions $HEMEti_1$ and $HEMEti_2$, whose speed is given by the following relation:

$$\lambda(HEMEti_1) = k_+\gamma V_t D\hat{W}_{HEMEti}; \qquad \lambda(HEMEti_1) = k_-\gamma V_t D\hat{W}_{HEMEti}$$

where V_t is the max velocity of the transport component that performs the uptake, k is the proportion of reversibility of heme transport reaction ($+$ for heme uptake; $-$ for heme efflux), and γ is the proportion of mass protein which is heme transporter. $D\hat{W}_{HEMEti}$ represents the average transporter amount of a *C. difficile* cell.

Subtraction of the endogenous uptake from the heme transport-induced uptake revealed a saturable component with an apparent V_t of 3.1 pmol/min/μ g protein (Shayeghi *et al.*, 2005). Given the average protein content of a bacterial cell around 55% of the dry weight and the average molecular weight of proteins in the cell is around 50 kDa, we compute the total number of proteins in bacterial cells assuming that the number of transporter proteins is a small fraction of the total number of proteins. Considering that the transporter protein has a similar weight to the average protein, then the mass of the transporter protein in a unit of bacterial mass can be calculated as the ratio of the number of transporter proteins to the total number of proteins in the cell.

C. difficile cell dynamics mudule.
This module models the proliferation response of a bacteria population upon treatment with antimicrobial therapy. This module is composed of 3 places: *CD*, *BiomassCD*, and *pheme_ c*. This module also include 4 transitions: *DeathBac*, *Dup*, *Starv* and *Detox*. The presence of a token in the place *CD* is interpreted as a biologically active *C. difficile* cell. *C. difficile* initial cell number was calculated from (Lynch *et al.*, 2013). Authors set the *in vitro* intestinal epithelial tissue as a planar IECs monolayer infected with a Multiplicity of Infection (MOI) of 100:1, *i.e.* 100 bacterial cells for each IEC. Given that we consider an initial number of IECs of $4.57 * 10^5$ cells, the initial number of *C. difficile* cells is $4.57 * 10^6$ cells.

The place *BiomassCD* indicates the average cellular biomass dry weight measured in *pg* per cell. *C. difficile* are Gram-positive rods, measuring 2-9 μm in length (a) and 0.3-0.7 μm in width (d) (Modaber, 1975). The cell shape shape can be approximated as a spherocylinder, a cylinder with hemispherical caps. Given the quoted diameter and length, we can compute a more refined estimate for the cellular biovolume of:

$$V_{CD,mean} = \frac{\pi d^2_{CD,mean}}{4}\left(\frac{4 * d_{CD,mean}}{6} + a_{CD,mean}\right) \approx 1.145\mu^3 \quad \text{(S22)}$$

We can compute a more refined estimate for the biomass per cell of:

$$DW_{CD,mean} = \frac{1}{3}V_{CD,mean}1.310^{-12} \approx 0.496e^{-12}g/cell. \quad \text{(S23)}$$

because most cells are about 2/3rd water, and the other components, like proteins, have a characteristic density of about 1.3 times the density of water.

The modelled system repurposes heme to counteract antimicrobial oxidative stress responses. The transition *Detox* the activity of hemoprotein HsmA to reduce oxidative damage caused by antibiotic action (Knippel *et al.*, 2020). This event models the suggestion that the HsmA protein interacts with heme and the drug, and then returns to its original state, presumably after having altered the heme and/or drug in some way to reduce their toxicity or reactivity. This is consistent with recent findings which suggest that HsmA uses heme to protect *C. difficile* from oxidative stress. HsmA is a protein that contains a heme prosthetic group where the intracellular heme molecule could be the replaceable cofactor. The kinetic analysis of the system led to the scheme:

$$\mathrm{PFe_3 + RO_2{}^{\bullet -} \longrightarrow [PFe_3] + RO_2{}^{-}} \tag{S24}$$

The rate constant for the reaction of radical reduction is equal to $1.5 * 10^{10}$ 1/mmol*min (Brault, 1985). The molarity of hemoprotein HsmA expressed by each bacterial cell is unknown. Therefore, the parameter r_{Detox} was defined by the model calibration phase.

The general transition *DeathBac* quantifies the clearance rate of bacterial cells by biomass minimum production. It is chosen according to the mean dry weight computed as shown in equation (S23). So the function is defined as:

$$f_{DeathBac}(\hat{x}(\nu), \nu) = \frac{h x_{CD}(\nu)}{2 + e^{x_{BiomassCD}(\nu) - DW_{CD,mean}}} \tag{S25}$$

where $DW_{CD,mean}$ represents one bacterial cell's mean observed dry weight. The parameter h is the death rate representing lifespan decline due to depletion of nutritional resources, at which time the cell growth rate slows.
The difference between the average biomass of a cell in the bacterial population and the mean biomass for a bacterial cell observed in nature. It measures of how much the average biomass of a cell in the population deviates from what is typically observed in nature. This difference could have various implications depending on the specific biological context. For example, a positive value could indicate that the bacterial population is well-fed or experiencing favourable conditions, allowing them to grow larger than average. On the other hand, a negative value could indicate that the bacterial population is under stress or experiencing unfavourable conditions, causing them to be smaller than average.

The transition *Dup* models bacterial cell proliferation based on exponential biomass accumulation computed in each iteration until a threshold is reached and the cell divides. *Dup* is a general transition whose velocity is defined by the following function:

$$f_{Dup}(\hat{x}(\nu), \nu) = r_{CDdup} x_{CD}(\nu) \frac{x_{BiomassCD}(\nu) - gDW_{CDmin}}{gDW_{CDmax} - gDW_{CDmin}} \left(1 - \frac{x_{CD}(\nu)}{CD_{max}}\right)$$

where r_{CDdup} is the doubling time, CD_{max} the theoretical maximum bacteria cell concentration, and gDW_{CDmax}/gDW_{CDmin} the maximum/minimum biomass dry weight. We assumed that the maximum population for *C. difficile* at the end of the growth phase can reach a density of 10 billion cells per mL (Bressloff, 2014). *C. difficile* is a slow-growing bacterium. The generation time of *C. difficile* can range from 27 minutes to 216 minutes, depending on the strain and conditions. If we consider r_{CDdup} as the duplication rate per hour, a reasonable estimate might be between 0.28 (for a 216-minute generation time) and 2.22 (for a 27-minute generation time) divisions per hour.

The transition *Starv* models the recognition that microorganisms satisfy their maintenance homeostasis consuming biomass. Its parameter is defined as $\lambda(Starv) = EX\_B\_starv$, defined as the biomass flow at which the biomass is consumed for maintenance. A growth rate of 0.30 (1/h) requires biomass production in the range of 0.14 (mmol/gDW*h) (as a validation test, it is verisimilar that such biomass production requires glucose uptake of about 30 mmol/gDW*h)

Metronizadole action mudule.
This module models the mechanism of action of the antibiotic causing an imbalance between oxidative and antioxidative processes, inducing oxidative stress and cell death. This module is comprehensive of 1 place, *Drug*, which quantifies the amount of intracellular antibiotic. The module includes 3 transition: *Death4Treat*, *Efflux* and *Treat*.

The reduction of the drug promotes the formation of intermediate compounds and toxic free radicals. Free radicals interact with intracellular targets, especially with host cell DNA resulting in DNA strand breakage and deadly destabilisation of the DNA helix. Therefore, The efficacy of antibiotic treatment is dependent on the antibiotic amount to which the bacterium is exposed and on the intrinsic bacteria strain features which influence its susceptibility to therapy.
Transition *Death4Treat* represents the efficacy of antibiotic treatment at killing bacteria. Input places for this transition are *Drug* and CD. The multiplicity of the corresponding input arc connecting CD to *Death4Treat* quantifies the bacteria cell number lost for each unit of drug. The antimicrobial killing rate is defined as:

$$f_{Death4Treat}(\hat{x}(\nu), \nu) = r_{Death4Treat} x_{CD}(\nu) x_{Drug}(\nu)\ e^{\frac{x_{Drug}(\nu)}{Drug_{MIC}}} \tag{S26}$$

where $r_{Death4Treat}$ represents the effectiveness of initial antibiotic therapy. The magnitude associated with this parameter was defined during the model calibration phase. The antibiotic concentration $Drug_{MIC}$ is the Minimum Inhibitory Concentration (MIC) of the modelled antibiotic. The rate increases as the ratio of the drug concentration to its MIC increases. This could model the increased efficacy of the drug as its concentration surpasses the MIC.

The transition *Efflux* models the antibiotic efflux pathways that can be present in some bacteria strains, and it is defined by the constant parameter $\lambda(Efflux) = efflux$, where $efflux$ represents the activity of Multi-Drug Efflux Transporter (MDET) that cause the efflux of intracellular antibiotics. We account kinetic parameters for the efflux pump from (Nagano and Nikaido, 2009). They cite a V_{max} of $2.35 * 10^{11}$ $mol/s/10^9 * cell$ which implies a total of $2.35 * 10^{-12}$ $(mol/10^9 * cell$ of transporter.

Fixing the frequency of daily dosing in the present study for simplicity, the transition *Treat* model the periodical antibiotic injections in the system. We designed the model to give an internal antibiotic concentration around the MIC, which implies a

sub-prescribed *dose* of 0.05 (g/day). Every 8 h of time simulation the transition *Treat* adds an amount of antibiotic measured as *pmol* to the *Drug* place equal to $\frac{\Delta_{drug} * 8 * dose}{24 * M_{mtz}}$, where M_{mtz} is the drug molecular weight (metronidazole), and Δ_{drug} is the scaling factor accounting for the amount of active drug reaching the site of action.

***C. difficile* metabolic network module.**
This module incorporates all metabolic reactions of the *C. difficile*. The metabolic level is connected with the *Lumen env module* by a set of 16 transitions. *EX_ biomass_ e_ in* and *EX_ biomass_ e_ out* coupled with the metabolic model exchange biomass reaction, and *EX_ amino_ L_ e_ in*, *EX_ amino_ L_ e_ out* coupled with the metabolic model exchange essential amino acid reactions. Transitions *sink_ pheme_ c_ in* and *sink_ pheme_ c_ out* represent connections of the reversible sink reaction for intracellular heme with the *pheme_ c* place included in the *C. difficile cell dynamics mudule.* This reaction is functionally replaced by filling the undefined gap where heme is consumed by non-metabolic cellular processes but needs to be metabolized.

FBA identifies steady-state flux rates measured as $mmol/(g * h)$ through a metabolic network, satisfying stoichiometric mass-balance as well as reaction direction's constraints (the reaction's flux activity represents the growth rate measured in $1/h$, (Adadi *et al.*, 2012)). Metabolites are absorbed or released at an estimated rate and converted into biomass computed under the exponential growth phase. Biomass accumulation is limited to the maximal cell weight to restrict growth to physiologically feasible conditions. During optimisation, the upper bound of the objective function is set accordingly:

$$f_{upper}(v_{EX\_biomass\_e}, \hat{x}(\nu)) = DW_{CD,max} - x_{BiomassCD}, \tag{S27}$$

where $DW_{CD,max}$ represents one bacterial cell's maximum observed dry weight.

To incorporate flux balance analysis (FBA), we established constraints for the reactions *EX_ cys_ L(e)*, *EX_ trp_ L(e)*, *EX_ val_ L(e)*, *EX_ ile_ L(e)*, *EX_ leu_ L(e)*, *EX_ pro_ L(e)*. The upper bounds for these reactions are set to zero, reflecting that there is no net production of aminoacids. The lower bound for each reaction, indicating the rate of metabolite consumption, is defined as follows:

$$f_{lower}(v_{EX\_m}, \hat{x}(\nu)) = -x_m(\nu)\frac{C}{x_{CD}(\nu)x_{BiomassCD}(\nu)}, \tag{S28}$$

where $x_m(\nu)$ represents the concentration of the metabolite corresponding of the reaction EX_m, and C is a conversion scalar that bridges the units of concentration to the flux units in the FBA model. The lower bound in FBA, expressed in mmol/gDW/h, denotes the rate of metabolite consumption or production normalized to the biomass. A negative value for the lower bound indicates the consumption of the metabolite by the organism. To correctly map biomass values into the flux framework, considering that 1 pg (picogram) of biomass corresponds to 1×10^{-12} grams, we compute the conversion scalar C as follows: $C = \left(\frac{N_a}{c \times 1 \times 10^{-12}}\right)$, where Na is the number of molecules per mmol. Each token assigned to amino acids' places counts c molecular units. Considering the reaction $sink\_pheme(c)$, its upper bound is set equal to 10, and its lower bound is defined as follows:

$$f_{lower}(v_{sink\_pheme(c)}, \hat{x}(\nu)) = -\frac{x_{pheme\_c}(\nu)1e-9}{x_{CD}(\nu)x_{BiomassCD}(\nu)1e-12}. \tag{S29}$$

here, we need to correctly map flux values as $pmol = 10^{-9}mmol$, analogously to S29. Finally, once the fluxes are estimated, the inverse functions of Eq.s S29 and S28 are applied to the correspondent v to convert it as a rate to associate with the transition.

Tables

Constant	Value	Reference
α	$0.95\ (unit)$	(Trommelen *et al.*, 2021)
β	$0.15\ (unit)$	(Monsen, 1988)
δ	$4.32274^{-11}\ (unit)$	estimated
Δ	$10^{-4}\ (unit)$	estimated
Na	$6.022^{20}\ (molecule)$	–
c	$6.022^{8}\ (molecule)$	–
DW_{IEC}	$10^{-9}\ (g/cell)$	(Halldorsson *et al.*, 2017)
G	$0.06\ (unit)$	–
D_v	$0.03\ (mm)$	(Kachlik *et al.*, 2010)
f_{trp}	$0.013\ (unit)$	(Dyer, 2010)
f_{cys}	$0.033\ (unit)$	(Dyer, 2010)
S_{pro}	$0.41248\ (unit)$	(Thiele *et al.*, 2013)
S_{leu}	$0.54554\ (unit)$	(Thiele *et al.*, 2013)
S_{ile}	$0.28608\ (unit)$	(Thiele *et al.*, 2013)
S_{val}	$0.35261\ (unit)$	(Thiele *et al.*, 2013)
S_{trp}	$0.013306\ (unit)$	(Thiele *et al.*, 2013)
S_{cys}	$0.046571\ (unit)$	(Thiele *et al.*, 2013)
M_{pro}	$0.11513\ (g/mmol)$	–
M_{leu}	$0.13117\ (g/mmol)$	–
M_{ile}	$0.13117\ (g/mmol)$	–
M_{val}	$0.117151\ (g/mmol)$	–
M_{trp}	$0.20423\ (g/mmol)$	–
M_{cys}	$0.12116\ (g/mmol)$	–
M_{heme}	$0.6165\ (g/mmol)$	–
d_{pheme_e}	$0.75\ (mg/(day * person))$	(He *et al.*, 2020)
e_{pheme_e}	$0.80\ (unit)$	(Monsen, 1988)
h	$0.01\ (cell/h)$	–
r_{CDdup}	$0.21\ (cell/h)$	(Curry, 2010)
CD_{max}	$10^{10}\ (cell/mL)$	(Bressloff, 2014)
EX_B_starv	$0.015\ (mmol/gDW * h)$	(Low and Chase, 1999)
$r_{Death4Treat}$	$6.475 \times 10^{-10}\ (1/h)$	estimated
r_{Detox}	$4.115 \times 10^{-5}\ (1/h)$	estimated
$r_{IECsDeath}$	$9.98 \times 10^{-9}\ (1/h)$	estimated
$Drug_{MIC}$	$10^{-6}\ (g/mL)$	(Lynch *et al.*, 2013)
$efflux$	$3.24^{-10}\ (1/cell * pmol * h)$	(Nagano and Nikaido, 2009)
M_{mtz}	$1.7116^{-10}\ (g/pmol)$	–
V_t	$3.1\ (pmol/min/gprotein)$	(Shayeghi *et al.*, 2005)
k_+	$0.05\ (unit)$	–
k_-	$1 - k_+\ (unit)$	–
γ	$10^{-3}\ (unit)$	–

Table S1. Selected constants of the dynamical model simulations. The columns correspond to the therapeutic conditions.

Place	Value	Reference
pro_L_v	0 $(mmol/mL)$	–
leu_L_v	0 $(mmol/mL)$	–
ile_L_v	0 $(mmol/mL)$	–
val_L_v	0 $(mmol/mL)$	–
trp_L_v	0 $(mmol/mL)$	–
cys_L_v	0 $(mmol/mL)$	–
IECs	4.57×10^5 (cell)	(Anonye *et al.*, 2019)
Damage	0 (cell)	–
pro_L_e	0.36 $(\mu mol/mL)$	(Adibi and Mercer, 1973)
leu_L_e	0.57 $(\mu mol/mL)$	(Adibi and Mercer, 1973)
ile_L_e	0.32 $(\mu mol/mL)$	(Adibi and Mercer, 1973)
val_L_e	0.58 $(\mu mol/mL)$	(Adibi and Mercer, 1973)
trp_L_e	0.098 $(\mu mol/mL)$	estimated
cys_L_e	0.25 $(\mu mol/mL)$	estimated
pheme_e	2×10^{-5} $(mmol/mL)$	(Hopp *et al.*, 2020)

Table S2. List of initial conditions of the dynamical model simulations.

Table S3. Parameter configurations identified through the median trajectory analysis

Configuration	Detox	Death4Treat	IECsDeath
Set 1	7.665760×10^{-4}	2.419458×10^{-10}	6.152626×10^{-9}
Set 2	7.113970×10^{-4}	5.419605×10^{-10}	3.912461×10^{-9}
Set 3	3.210076×10^{-4}	2.088699×10^{-10}	1.546280×10^{-9}

Figures

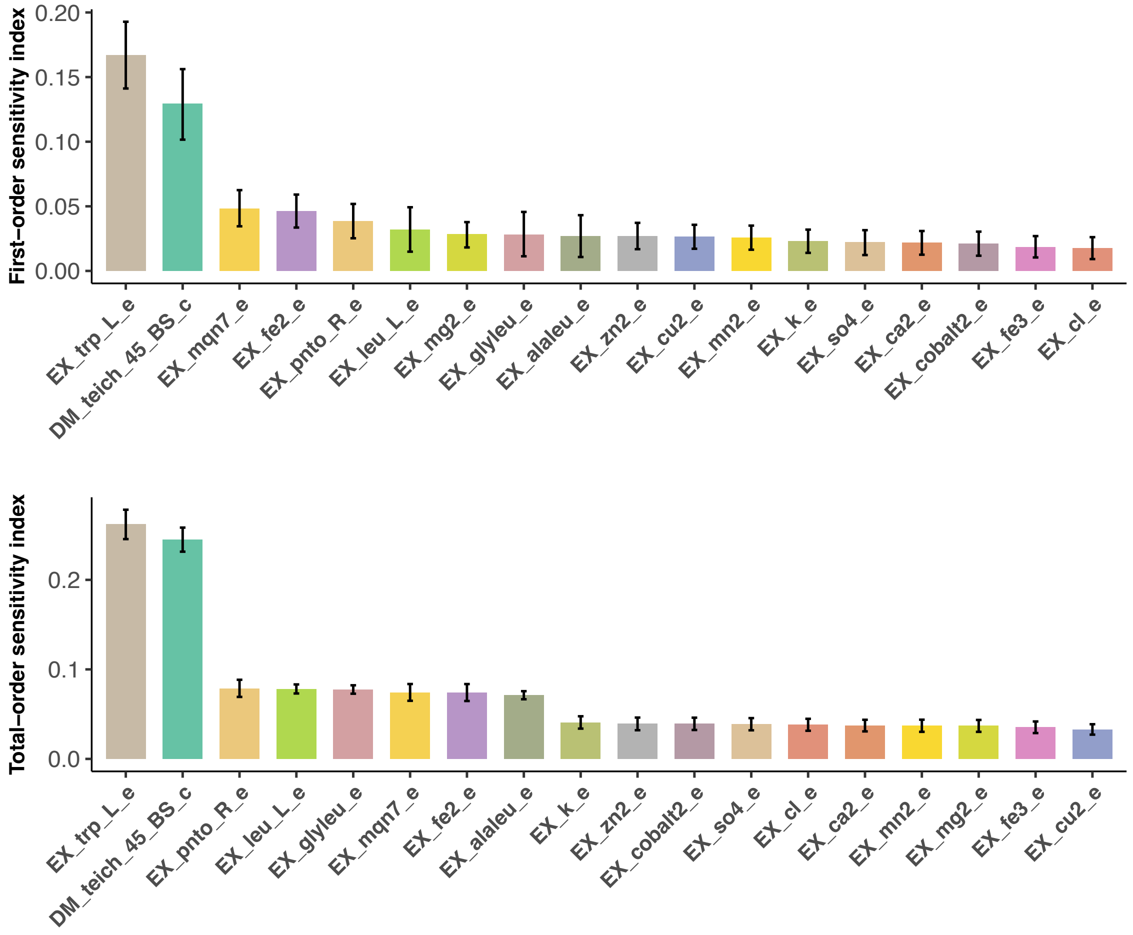

Fig. S1. Results of the global SA on the *C. difficile* metabolic model. First-order sensitivity indices (top) and the total effect indices (bottom) and their low and high confidence levels. Bootstrapping is used to estimate the sampling distribution and to construct bootstrap confidence intervals on sensitivity indices. Sensitivity coefficients below 0.01 were considered negligible.

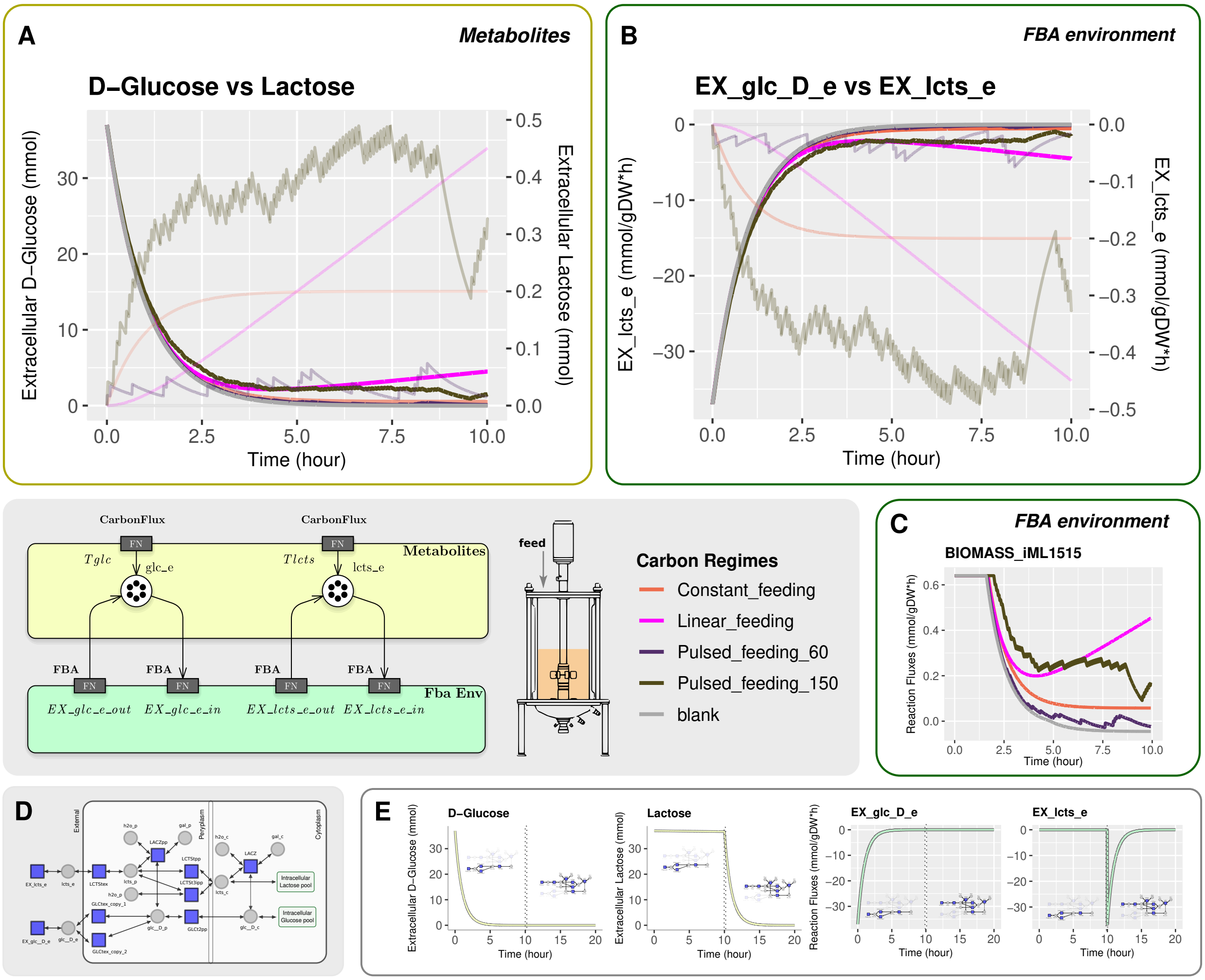

Fig. S2. Overview of results for the *E. coli* batch growing model according to the supplementation scenario over time. (A) Profiles representing the time-course changes in the concentrations of D-glucose and lactose under different conditions of carbon administration. (B) FBA-estimated metabolic fluxes for D-glucose and lactose exchanges at time resolution. (grey panel) Petri Net model and schematic diagram of a bioreactor, (C) FBA-estimated *E. coli* biomass objective function at time resolution. (D) Graph of the subnetwork from iML1515 for the uptake of D-glucose and Lactose and components encoded by the lac operon. Grey circles represent the metabolites from the subnetwork, and blue squares represent the subsystem reactions. The pathway starts with the transport of D-Glucose and Lactose from the extracellular space to the periplasm, where lactose can be transformed into periplasmatic D-Glucose (glc_D_p) through a reaction from periplasmatic beta-galactosidase. D-glucose and lactose can also be directed to the cytoplasm where beta-galactosidase cleaves lactose into D-glucose. (E) Results collectively highlight the role of the LacZ system in enabling the switch from D-glucose to lactose metabolism when necessary.

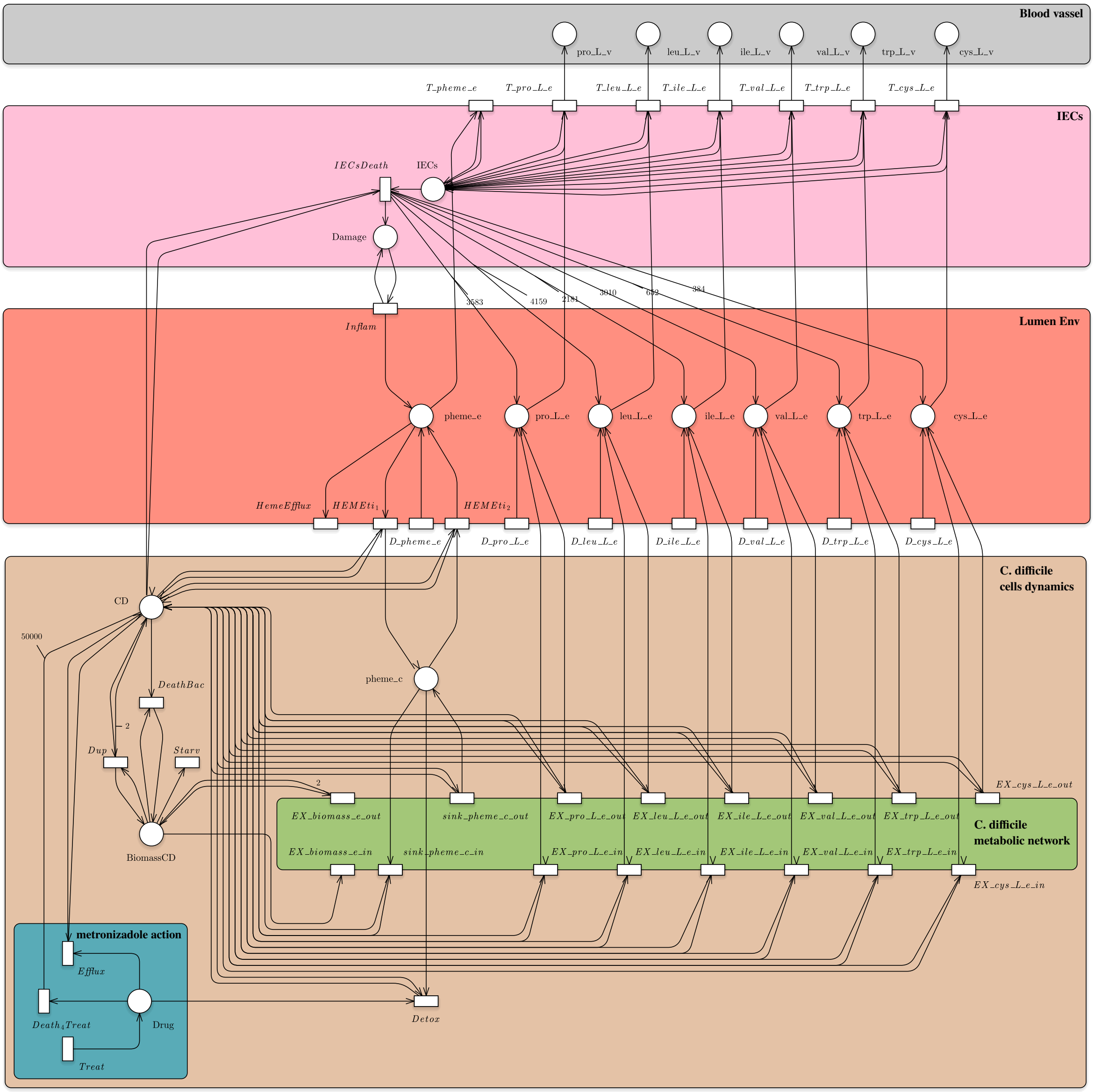

Fig. S3. The ESPN associated with the CDI model is composed of places (graphically represented by circles) corresponding to epithelial cells, bacterial biomass, metabolites and tissue state (i.e. damage to the colonic mucosa), and of transitions (graphically represented by rectangles) representing interactions among the entities, cellular death, intake or efflux of metabolites, toxin action, intestinal inflammation and drug activity.

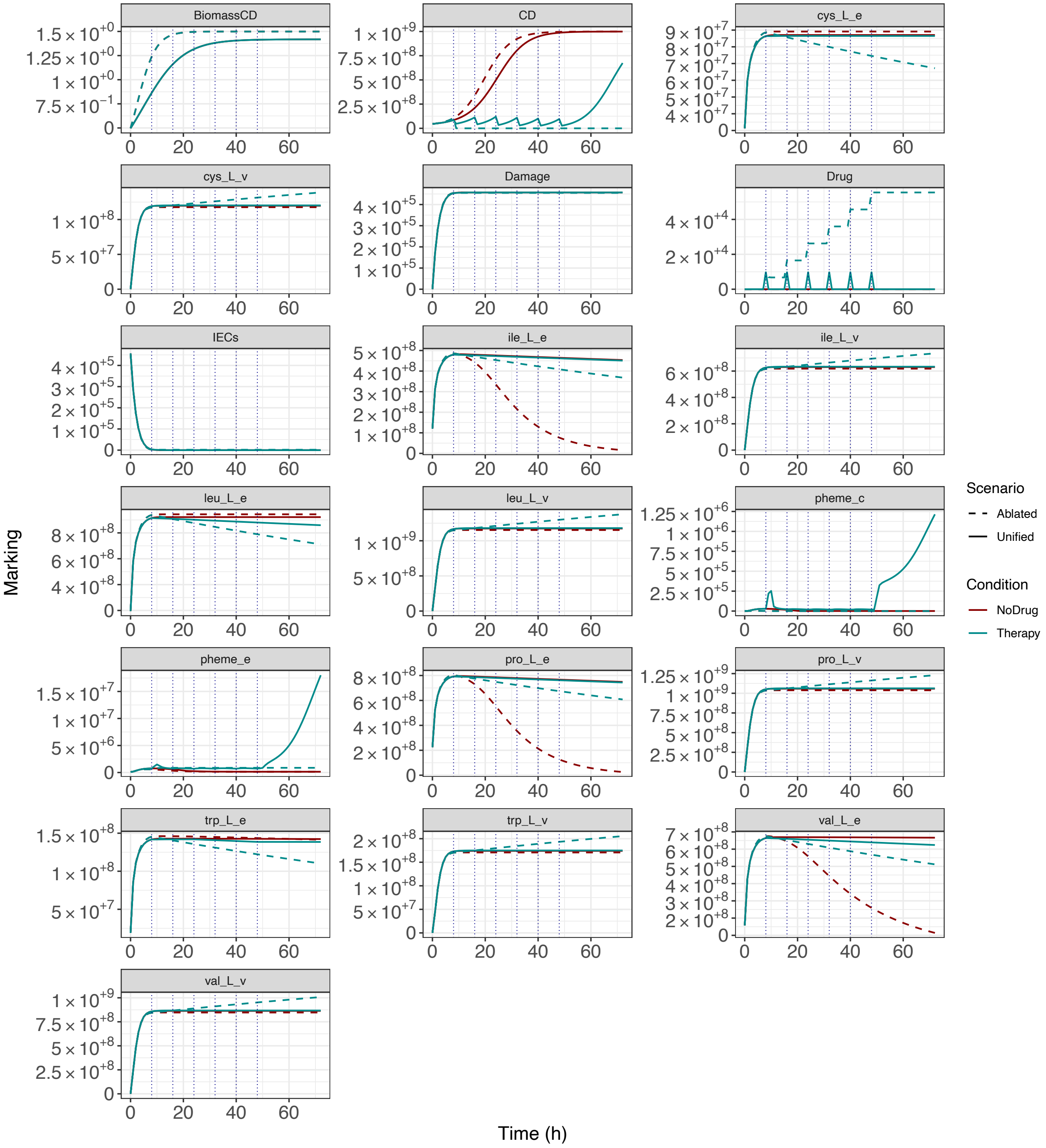

Fig. S4. The dynamic behaviour of the principal places of the ESPN model given two scenarios, untreated and treatment conditions (red and blue colours, respectively), and the two approaches, unified and ablated (solid and dashed lines, respectively).

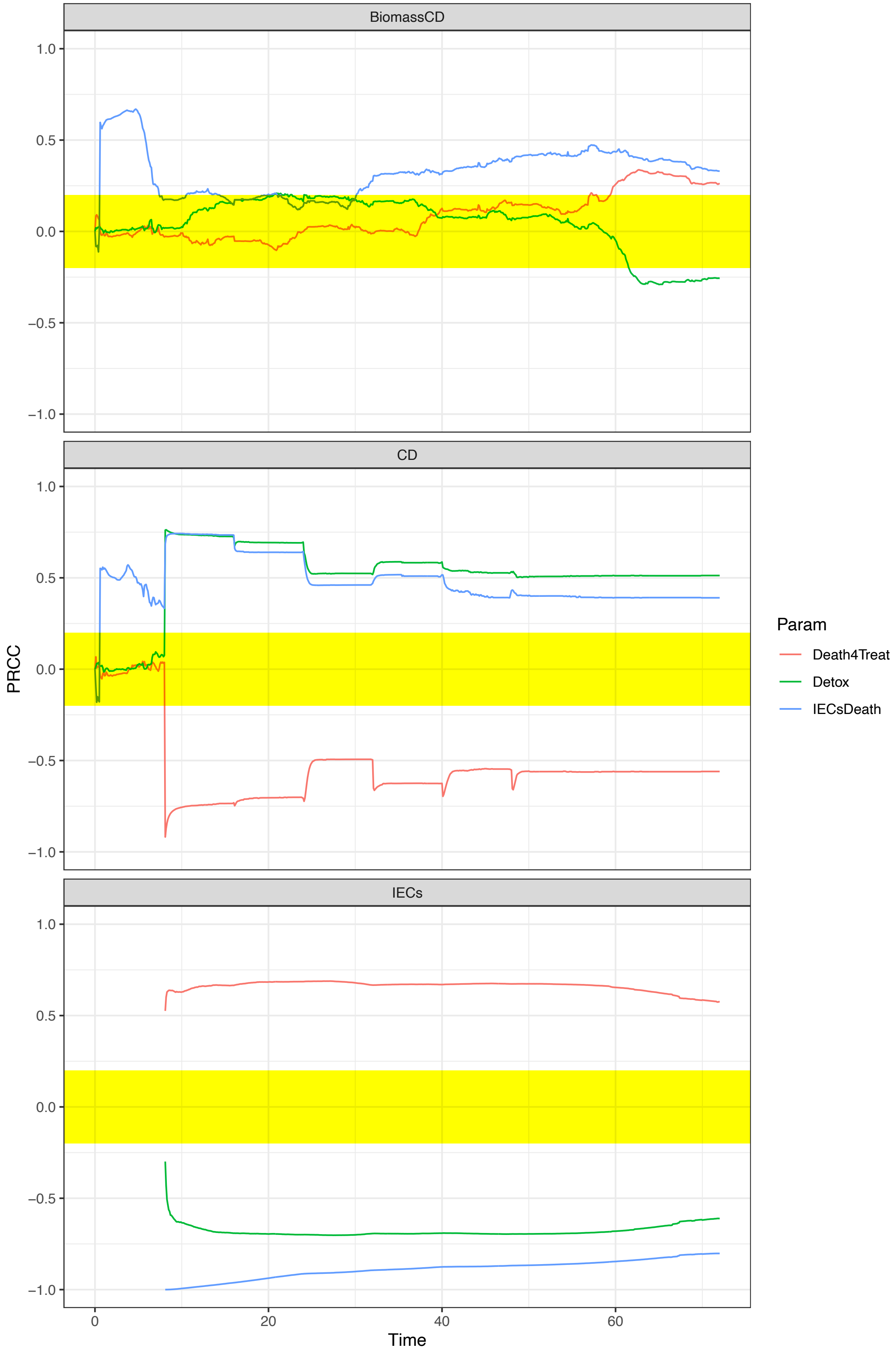

Fig. S5. PRCCs over the whole time interval for each model parameter is reported. Yellow area represents the zone of non-significant PRCC values

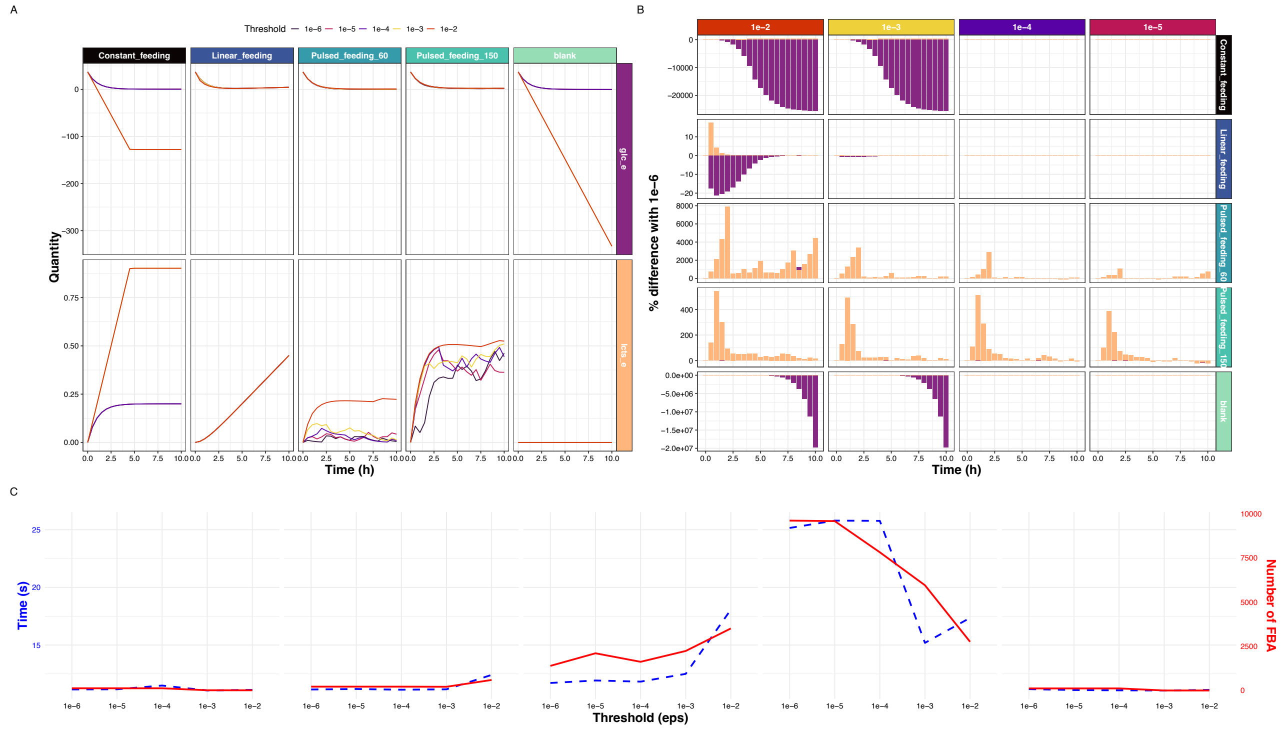

Fig. S6. Performance of the E. coli model.

Performance Evaluation

The computational cost of our framework is indeed dependent on the size of the metabolic network and the ODEs system. Furthermore, as explained in Section S1.5, another dependency is given by the threshold criterion that compares the elements of the ODEs affecting the FBA at two consecutive solution times which improves the model's efficiency by avoiding superfluous computations. Note that the performance obtained by studying the two models shown in this paper is taken by solving the unified model using the *GreatMod* framework in the High-Performance Computing for Artificial Intelligence data centre (`https://hpc4ai.unito.it`) (Aldinucci *et al.*, 2018). The computational cost comprises the sum of two components: (i) the time required to solve the coupled ODE system with its corresponding FBA problem, and (ii) the Docker container execution overhead.

Escherichia coli unified model

Let us consider the *E. coli* model introduced in section S2. The top-left plot in figure S6 illustrates the dynamics over time of the two places of the *E. coli* PN model (glc_e and $lcts\_e$), representing the changes in the concentrations of D-glucose and lactose under the five different conditions of carbon administrations (constant feeding, linear feeding, pulsed feeding 60, pulsed feeding 150, and blank) and with different threshold values (from $1e^{-6}$ to $1e^{-2}$).

The top-right plot shows the percentage difference in the dynamics when varying the threshold tolerance values compared to a baseline of $1e^{-6}$. As the tolerance increases, the percentage difference grows, reflecting a loss in precision. This trend is expected since larger threshold values permit greater approximation errors. Scenarios like pulsed feeding appear more sensitive to changes in epsilon compared to constant or linear feeding, likely due to their more complex dynamics. Furthermore, it is possible to see that with high threshold tolerance values such as $1e^{-2}$, some dynamics of the glc_e place become negative. This is due to rates too high of the transitions that remove elements from glc_e place, which needs to be updated more frequently.
The bottom plot focuses on the computational impact of changing threshold values. An increase in the threshold parameter results in fewer FBA solver calls, thereby reducing the computational time required for solving the ODE system due to the implementation of more relaxed numerical tolerances. Generally, we can observe that the computational cost is not too high, from a few seconds to 20 seconds for the more complex simulations of the "Pulsed_feeding_150" scenario.

Clostridium difficile infection

The figure S7 shows the performance obtained by simulating the *C. difficile* strain CD196 model introduced in Section S3 and in the main paper 3, considering different threshold values, the three-parameter configuration sets and the unified experiment setting (since in the ablated and partial ablated the FBA calculation does not depend on the threshold tolerance value). Similarly to the figure S6, as the tolerance increases, the percentage difference in the marking of the model places w.r.t. the baseline of $1e^{-6}$ grows. In this context, we showed only the four places with the highest difference for simplicity.
The lower plot highlights the computational impact of varying threshold values within the unified experiment setting, as it is the

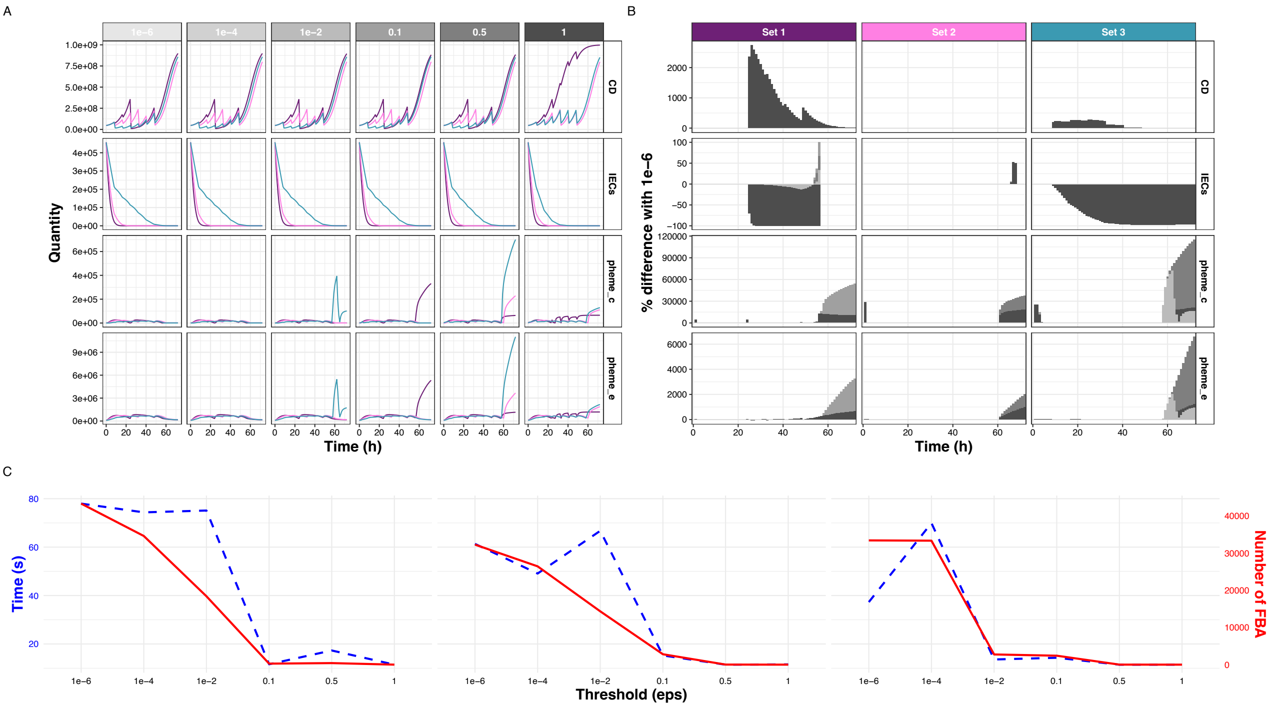

Fig. S7. Performance of the C. difficile model, considering the unified experiment setting.

sole scenario where the FBA is solved in conjunction with the ODEs system. Generally speaking, the computational expense for this model remains modest, peaking at 80 seconds. Notably, the computational cost of the ablated experiment setting (where the FBA is solved only initially) averages around 12 seconds. Consequently, the unified setting is not significantly more demanding in terms of computation compared to the simpler ablated setting.

In summary, the results emphasize the balance between computational efficiency and simulation accuracy. Lower threshold values provide precise results but require more computational resources. Thus, this shows a clear trade-off between precision and efficiency. Furthermore, we can observe from these results that, although increasing the threshold tolerance reduces the number of FBA calls, the overall computational cost does not decrease proportionally. This is because the total cost is influenced not only by the FBA resolution but also by the time required to solve the ODE system and the Docker execution time, both of which may remain relatively constant or be less affected by changes in tolerance.

Supplementary References

[supp1]Adadi, R. *et al.* (2012). Prediction of microbial growth rate versus biomass yield by a metabolic network with kinetic parameters. *PLoS computational biology*, **8**, e1002575.

[supp2]Adibi, S. and Mercer, D. (1973). Protein digestion in human intestine as reflected in luminal, mucosal, and plasma amino acid concentrations after meals. *The Journal of clinical investigation*, **52**, 1586–94.

[supp3]Aldinucci, M. *et al.* (2018). HPC4AI: an AI-on-demand federated platform endeavour. In *Proceedings of the 15th ACM International Conference on Computing Frontiers*, pages 279–286.

[supp4]Ammar, E. *et al.* (2018). Regulation of metabolism in escherichia coli during growth on mixtures of the non-glucose sugars: Arabinose, lactose, and xylose. *Scientific Reports*, **8**.

[supp5]Amparore, E. *et al.* (2016). *30 years of greatSPN*, pages 227–254.

[supp6]Anonye, B. *et al.* (2019). Probing clostridium difficile infection in complex human gut cellular models. *Frontiers in Microbiology*.

[supp7]Argiles, J. and Lopez-Soriano, F. (1990). Intestinal amino acid transport: An overview. *The International journal of biochemistry*, **22**, 931–7.

[supp8]Bergholz, T. *et al.* (2007). Global transcriptional response of escherichia coli o157:h7 to growth transitions in glucose minimal medium. *BMC microbiology*, **7**, 97.

[supp9]Brault, D. (1985). Model studies in cytochrome p-450-mediated toxicity of halogenated compounds: radical processes involving iron porphyrins. *Environmental Health Perspectives*, **64**, 53–60.

[supp10]Bressloff, P. C. (2014). *Stochastic processes in cell biology*, volume 41. Springer.

[supp11]Castagno, P. *et al.* (2020). A computational framework for modeling and studying pertussis epidemiology and vaccination. *BMC bioinformatics*, page 21:344.

[supp12]coli Genome Project University of Wisconsin–Madison, E. (2023). Publications. Accessed: 14-Nov-2023.

[supp13]Colom, J. and Silva, M. (2006). *Convex geometry and semiflows in P/T nets. A comparative study of algorithms for computation of minimal p-semiflows*, pages 79–112.

[supp14]Covert, M. *et al.* (2008). Integrating metabolic, transcriptional regulatory and signal transduction models in escherichia coli. *Bioinformatics*, **24**(18), 2044–2050.

[supp15]Covert, M. W. and Palsson, B. Ø. (2002). Transcriptional regulation in constraints-based metabolic models of escherichia coli. *Journal of Biological Chemistry*, **277**(31), 28058–28064.

[supp16]Curry, S. (2010). Clostridium difficile. *Clinics in laboratory medicine*, **30**, 329–42.

[supp17]Debast, S. *et al.* (2014). European society of clinical microbiology and infectious diseases: Update of the treatment guidance document for clostridium difficile infection. *Clinical Microbiology and Infection*, **20**.

[supp18]Dingsdag, S. and Hunter, N. (2017). Metronidazole: an update on metabolism, structure–cytotoxicity and resistance mechanisms. *Journal of Antimicrobial Chemotherapy*, **73**.

[supp19]Dyer, K. (2010). The quiet revolution: A new synthesis of biological knowledge. *Journal of Biological Education*, **February 1971**, 15–24.

[supp20]Edwards, A. *et al.* (2014). Conserved oligopeptide permeases modulate sporulation initiation in clostridium difficile. *Infection and immunity*, **82**.

[supp21]Gutierrez, M. *et al.* (2021). Characterizing bulk rigidity of rigid red blood cell populations in sickle-cell disease patients. *Scientific Reports*, **11**, 7909.

[supp22]Halldorsson, S. *et al.* (2017). Metabolic re-wiring of isogenic breast epithelial cell lines following epithelial to mesenchymal transition. *Cancer Letters*, **396**.

[supp23]Hao, T. *et al.* (2010). Compartmentalization of the edinburgh human metabolic network. *BMC bioinformatics*, **11**, 393.

[supp24]He, J. *et al.* (2020). Dietary nonheme, heme, and total iron intake and the risk of diabetes in adults: Results from the china health and nutrition survey. *Diabetes Care*, **43**, dc192202.

[supp25]Herajy, M. *et al.* (2018). Coloured hybrid petri nets: an adaptable modelling approach for multi-scale biological networks. *Computational Biology and Chemistry*, **76**.

[supp26]Hopp, M.-T. *et al.* (2020). Heme determination and quantification methods and their suita-bility for practical applications and every-day-use. *Analytical Chemistry*, **XXXX**.

[supp27]Kachlik, D. *et al.* (2010). The spatial arrangement of the human large intestinal wall blood circulation. *Journal of anatomy*, **216**(3), 335–343.

[supp28]Karasawa, T. *et al.* (1995). A defined growth medium for clostridium difficile. *Microbiology (Reading, England)*, **141 (Pt 2)**, 371–5.

[supp29]Karp, P. D. *et al.* (2018). How accurate is automated gap filling of metabolic models? *BMC systems biology*, **12**(1), 1–11.

[supp30]Knippel, R. *et al.* (2018). Heme sensing and detoxification by hatrt contributes to pathogenesis during clostridium difficile infection. *PLoS Pathogens*, **14**.

[supp31]Knippel, R. *et al.* (2020). Clostridioides difficile senses and hijacks host heme for incorporation into an oxidative stress defense system. *Cell Host and Microbe*, **28**.

[supp32]Kulkarni, N. *et al.* (2018). Reproducible bioinformatics project: A community for reproducible bioinformatics analysis pipelines. *BMC Bioinformatics*, **19**, 211–219.

[supp33]Liu, M. *et al.* (2005). Global transcriptional programs reveal a carbon source foraging strategy by. *The Journal of biological chemistry*, **280**, 15921–7.

[supp34]Low, E. W. and Chase, H. A. (1999). The effect of maintenance energy requirements on biomass production during wastewater treatment. *Water Research*, **33**(3), 847–853.

[supp35]Lularevic, M. *et al.* (2019). Improving the accuracy of flux balance analysis through the implementation of carbon availability constraints for intracellular reactions. *Biotechnology and Bioengineering*, **116**.

[supp36]Lynch, T. *et al.* (2013). Characterization of a stable, metronidazole-resistant clostridium difficile clinical isolate. *PloS one*, **8**(1), e53757.

[supp37]Magnusdottir, S. *et al.* (2016). Generation of genome-scale metabolic reconstructions for 773 members of the human gut microbiota. *Nature Biotechnology*, **35**.

[supp38]Marsan, A. *et al.* (1995). *Modelling with Generalized Stochastic Petri Nets*. J. Wiley, New York, NY, USA.

[supp39]Modaber, I. (1975). Clostridium difficile. *Acta Medica Iranica*, **18**(3-4), 111–128.

[supp40]Monsen, E. (1988). Iron nutrition and absorption: Dietary factors which impact iron bioavailability. *Journal of the American Dietetic Association*, **88**, 786–90.

[supp41]Nagano, K. and Nikaido, H. (2009). Kinetic behavior of the major multidrug efflux pump acrb of escherichia coli. *Proceedings of the National Academy of Sciences*, **106**(14), 5854–5858.

[supp42]Neumann-Schaal, M. *et al.* (2015). Time-resolved amino acid uptake of clostridium difficile 630Δerm and concomitant fermentation product and toxin formation. *BMC Microbiology*, **15**.

[supp43]Noronha, A. *et al.* (2018). The virtual metabolic human database: integrating human and gut microbiome metabolism with nutrition and disease. *Nucleic acids research*, **47**.

[supp44]Papoutsakis, E. T. (2000). Equations and calculations for fermentations of butyric acid bacteria. *Biotechnology and Bioengineering*, **67**(6), 813–826.

[supp45]Pernice, S. *et al.* (2019). A computational approach based on the colored petri net formalism for studying multiple sclerosis. *BMC bioinformatics*.

[supp46]Puy, A. *et al.* (2022). sensobol : An r package to compute variance-based sensitivity indices. *Journal of Statistical Software*, **102**.

[supp47]Qian, G. and Mahdi, A. (2020). Sensitivity analysis methods in the biomedical sciences. *Mathematical Biosciences*, **323**, 108306.

[supp48]Saltelli, A. *et al.* (2010). Variance based sensitivity analysis of model output. design and estimator for the total sensitivity index. *Computer Physics Communications*, **181**, 259–270.

[supp49]Shayeghi, M. *et al.* (2005). Identification of an intestinal heme transporter. *Cell*, **122**, 789–801.

[supp50]Solis, F. *et al.* (2005). Effect of prebiotic on gut development and ascites incidence of broilers reared in a hypoxic environment. *Poultry science*, **84**, 1092–100.

[supp51]Thiele, I. *et al.* (2013). A community-driven global reconstruction of human metabolism. *Nature biotechnology*, **31**, 419–425.

[supp52]Trommelen, J. *et al.* (2021). Gut amino acid absorption in humans: Concepts and relevance for postprandial metabolism. *Clinical Nutrition Open Science*, **36**.

[supp53]Varma, A. and Palsson, B. (1994). Stoichiometric flux balance models quantitatively predict growth and metabolic by-product secretion in wild-type escherichia coli w3110. *Applied and environmental microbiology*, **60**(10), 3724–3731.

[supp54]Voit, E. O. *et al.* (2015). 150 years of the mass action law. *PLoS computational biology*, **11**(1).

[supp55]W. Covert, M. *et al.* (2001). Regulation of gene expression in flux balance models of metabolism. *Journal of Theoretical Biology*, **213**(1), 73 – 88.

[supp56]Watson, M. R. (1984). Metabolic maps for the Apple II. *Biochemical Society Transactions*, **12**(6), 1093–1094.
